# A comprehensive pharmacological survey across heterogeneous patient-derived glioblastoma stem cell models

**DOI:** 10.1016/j.isci.2026.115839

**Published:** 2026-04-27

**Authors:** Richard J.R. Elliott, Peter W.K. Nagle, Muhammad Furqan, John C. Dawson, Vanessa Smer-Barreto, Diego A. Oyarzún, Aoife McCarthy, Alison F. Munro, Camilla Drake, Gillian M. Morrison, Steven M. Pollard, Michael Marand, Daniel Ebner, Valerie G. Brunton, Margaret C. Frame, Neil O. Carragher

**Affiliations:** 1 Cancer Research UK Scotland Centre (Edinburgh), Institute of Genetics and Cancer, University of Edinburgh, Western General Hospital, Edinburgh, UK; 2 School of Informatics, University of Edinburgh, 10 Crichton Street, Edinburgh EH8 9AB, UK; 3 School of Biological Sciences, University of Edinburgh, Max Born Crescent, Edinburgh EH9 3BF, UK; 4 Institute of Regeneration and Repair, Cancer Research UK Scotland Centre, University of Edinburgh, Edinburgh EH16 4UU, UK; 5 Nuffield Department of Medicine, University of Oxford, Old Road Campus, Oxford OX3 7BN, UK

**Keywords:** biological sciences

## Abstract

The lack of advancement in the treatment of glioblastoma (GBM) over the past two decades calls for more innovation to address the inter- and intra-patient heterogeneity confounding modern target-directed drug discovery strategies. In this study, we incorporate a panel of patient-derived GBM stem cell lines into an automated and unbiased “cell painting” assay to quantify multiple GBM stem cell phenotypes. By screening several compound libraries, followed by dose-response validation of hit compounds, we present a comprehensive survey of distinct pharmacological classes and druggable targets upon multiple GBM stem cell phenotypes. We further characterize two validated target classes, histone deacetylase and cyclin dependent kinase inhibitors. We demonstrate that unbiased Cell Painting phenotypic screening is a productive approach to identifying new targets, drug classes and future drug combinations that address the heterogeneity of GBM. We provide all GBM cell painting data for each compound perturbation for the research community to explore further.

## Highlights

• Cell painting assay applied across patient-derived glioblastoma stem cell line panel
• 3,866 compounds screened across six well-characterized glioblastoma stem cell lines
• Validated 211 compounds identifying multiple drug-target associations with GBM
• Mechanistic characterization of HDAC and CDK9 inhibitor hit classes in GBM assays

## Graphical abstract

**Figure:**
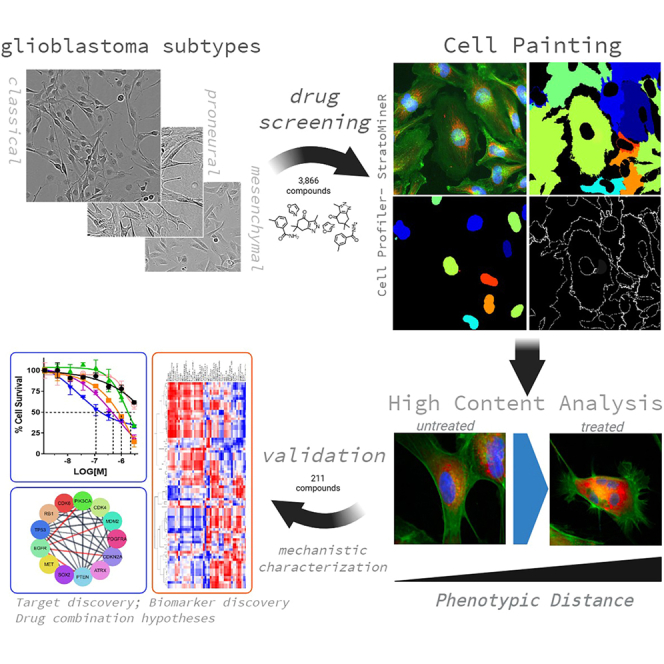

## Introduction

Glioblastoma (GBM) is a highly complex heterogeneous disease that remains difficult to treat. Mortality rates have increased over the last 30 years, especially in the over 60 age group, as incidence rates outpace survival improvements.^1^ Since the introduction of post-surgical, concomitant radiotherapy and adjuvant temozolomide in 2005 (the “Stupp protocol”), which increased median overall survival (mOS) by ∼3 months,^2^ a lack of effective, novel treatments for GBM have been introduced into broad clinical use and 5-year survival rates from time of diagnosis remain at less than 5%.^3^ Clinical and scientific GBM research over the last 20 years has revealed a complex landscape of remarkable interpatient, intratumoral and spatial heterogeneity.^4^^,^^5^^,^^6^^,^^7^^,^^8^^,^^9^ Early efforts to classify GBM heterogeneity through bulk transcriptomic profiling identified three GBM subtypes mesenchymal (MES), classical (CLA), and proneural (PRO),^6^^,^^10^^,^^11^ while subsequent single cell RNA sequencing revealed four types of malignant cellular states within isocitrate dehydrogenase (IDH) wild-type GBM tumors: MES-like, astrocytic-like, oligodendrocyte precursor and neural progenitor cell-like. Importantly all of these states co-exist within the same GBM tumor and can interconvert into each other.^12^ More recently, Nomura et al. identified three novel malignant cell states: a neuronal-like state, a glial progenitor cell-like state, and a cilia-like state.^13^ The complex biology driving the disease is further exacerbated by the addition of GBM stem cells (GSCs). The cancer stem cell hypothesis proposes that a small subset of cancer cells which have stem-cell-like properties, such as the ability to self-renew indefinitely, are responsible for tumor initiation, growth, metastasis, and recurrence.^14^ These cancer stem cells are common in brain tumors such as GBM and are resistant to standard anti-cancer treatments targeting rapidly dividing cells.^8^^,^^9^^,^^15^^,^^16^^,^^17^^,^^18^ More recent molecular GBM classification studies provide further resolution of multiple GSC sub-states. Garofano et al. proposed an alternative pathway-based classification system, which integrates multi-omics data to define GSC subtypes based on core biological functions rather than isolated gene expression patterns. These studies identified a further four cellular states: mitochondrial, glycolytic/plurimetabolic, neuronal, and proliferative/progenitor.^19^ Recent work by Greenwald et al. revealed GSC states are spatially patterned and shaped by local microenvironments.^20^ The ability of GSCs to transition between malignant cell states contributes to therapeutic resistance and relapse, limiting durable responses based on any personalized treatment strategies, including any initial GBM subtype/cell state classification.^21^^,^^22^ Phospho-proteomic studies performed on patient derived GBM model systems demonstrate rapid rewiring of pathway signaling at the post-translational pathway level following effective inhibition of some of the most compelling drug targets such as the mTOR pathway, which is hyperactivated in approximately 90% of GBM.^23^ GBM plasticity and heterogeneity thus confound modern target-based drug discovery (TDD) strategies contributing to drug resistance and relapse, hence the targeting of clear oncogenic drivers of GBM, such as EGFR amplification/mutation or RAS, PI3K signaling, has not translated into significant efficacy in GBM clinical management.

Phenotypic drug discovery (PDD), defined as the identification of hit or lead compounds prior to target identification,^24^ has historically contributed more than TDD, in terms of first-in-class drugs approved by the FDA since 1999,^25^^,^^26^ yet TDD has dominated drug discovery approaches in oncology over the same period of time.^27^ The drug development of imidazotetrazines, such as temozolomide, extended from chemocentric and phenotypic-led collaborative research exploring the *in vitro* and *in vivo* anti-cancer properties of small molecules, dacarbazine and mitozolomide (azolastone), emphasizing the value of incorporating phenotypic response data into early stage drug design in complex disease areas such as GBM. While TDD and PDD are complimentary drug discovery strategies, PDD can offer some advantages over target-based methods, as it allows discovery of potential therapeutics using disease relevant biological models of disease states where target biology is poorly understood.^28^^,^^29^ This “target-agnostic” approach has been greatly assisted by hardware advances in high-content microscopy, the development of advanced image analysis software tools and improved phenotypic profiling techniques, such as cell painting.^30^^,^^31^^,^^32^^,^^33^ The cell painting assay multiplexes six fluorescent dyes, imaged in five spectral channels to reveal eight broadly relevant cellular components or organelles. This can be combined with the development of bespoke automated image analysis pipelines to segment individual cells and calculate hundreds to thousands of morphological feature-based measurements per cell (e.g., various measures of size, shape, texture, intensity, and many others) to produce a data-rich profile that is suitable for the detection of subtle cellular phenotypes which can be linked to biological outcomes.^34^ Here we describe what is among the first examples of the adaptation and optimization of cell painting for profiling compound mechanism-of-action (MOA) and target-class across a panel of genetically distinct patient-derived GSC lines. Using this approach we provide a comprehensive pharmacological audit of how specific targets, pharmacological, and chemical classes influence patient-derived GBM cellular phenotypes. We present a large-scale application of this technique using chemically diverse libraries containing FDA-approved drugs, or phase I-passed clinical candidates and selective chemical probe compounds against known oncology targets. We identify multiple diverse pharmacological and target classes effective across our genetically distinct panel of patient-derived GSCs, including many not previously identified as potential GBM therapeutics, and present all validated screening hits and their target assignments to the GBM research community. These results support our hypothesis that an unbiased phenotypic led approach, using disease relevant patient-derived GSC models and exploitation of deep phenotyping and drug MOA profiling technologies, is a productive approach to identifying novel targets, drug classes, and future drug combinations that address the heterogeneity of GBM.

## Results

### Glioblastoma stem cell models

Patient-derived GSCs were obtained from the Cancer Research UK Glioma Cellular Genetics Resource, and six GSC lines were initially selected to cover common subtypes based on bulk transcriptomic subtype classification (CLA, MES, and PRO), (Figure S1A). Initial studies demonstrate compatibility with high-content imaging (i.e., flat and adherent morphology) and cell number was optimized for 384-well microtiter plate formats to enable high-throughput drug screening (Figures S1B–S1D). In order to begin characterizing these cell lines, prior to phenotypic screening we examined basal protein expression and post-translational pathway activation status by reverse phase protein array (RPPA) and cytokine array in 2-dimensional (2D) and 3-dimensional (3D) cell culture conditions. Basal levels of protein and phospho-epitope abundance (in both 2D and 3D cultures) show functional enrichment of signaling pathways in MAPK, ErbB, VEGF, PI3K-AKT, focal adhesion, EGFR inhibitor resistance, and PD-1 checkpoint in cancer (Figures S2A and S2B). The cytokine array profiling further demonstrates enrichment in the secretion of GBM associated chemokine proteins involved in tumor progression, invasion, angiogenesis (IL-6, MMP-7, TIMP1, and VEGF), and tumor-associated neutrophils (TANs; CCL2, and IL-8/CXCL8)^35^ including factors previously shown to contribute to GSC subtype *trans*-differentiation (PRO to MES transition)^36^ (Figures S2C and S2D). Notably the six GSC lines representing three pairs of the common GBM transcriptomic sub-types (CLA, MES, and PRO) selected for our study do not cluster into any specific subgroups based on basal protein expression or pathway activation status. Interestingly, high levels of the pro-inflammatory cytokine, interleukin-8 (CXCL8, Figures S2C and S2D) have recently been reported as a druggable target in GBM (via humanized anti-IL8 antibody) with an anti-PD-1 combinational blockade.^37^ Overall, this pre-screening profiling indicates our proposed GSC drug screening models are representative of typical GBM signaling networks observed in patient samples, express known druggable pathways while capturing the expected broad heterogeneity of GBM tumors at the molecular, protein and morphological levels in both 2D adherent and 3D cell culture.

### GSC cell painting assay development

**Figure 1:**
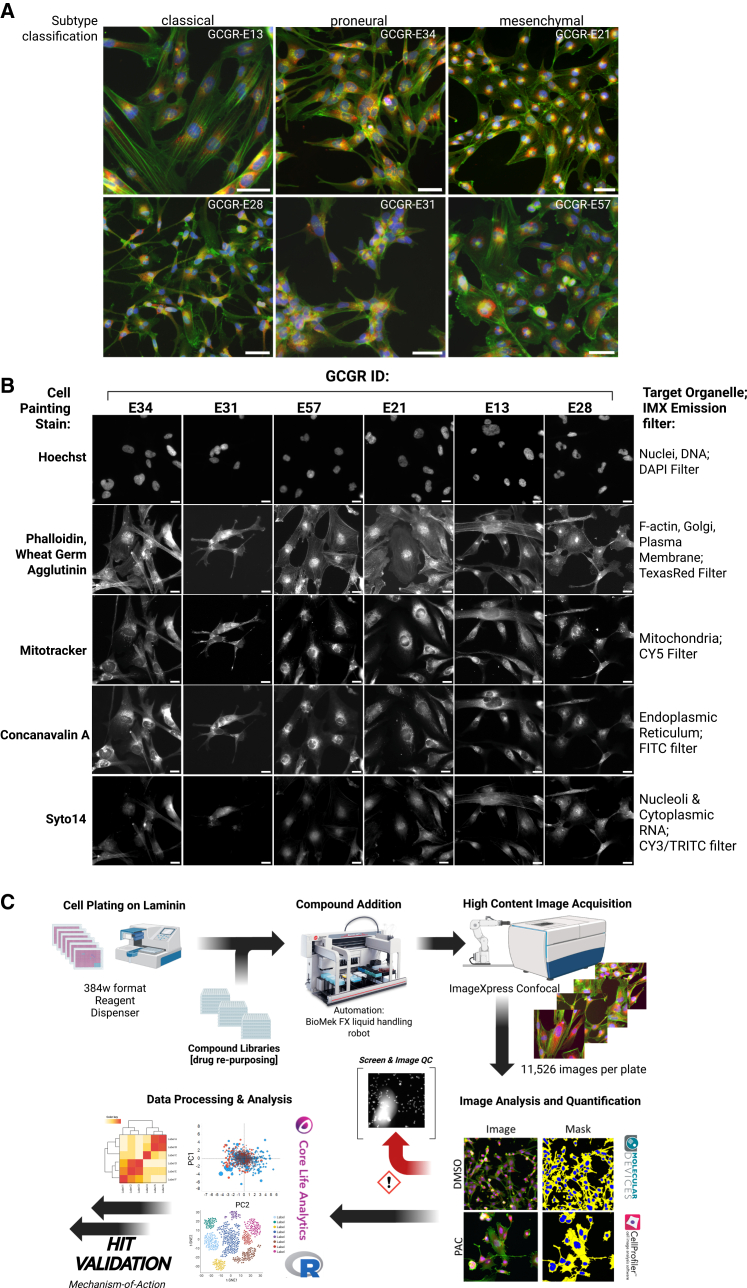
High content phenotypic assay screening on GSC panel (A) Representative images of cell painting across GSC panel (Hoechst, blue; Phalloidin/WGA, green; endoplasmic reticulum, red). Scale bars, 50 μm. (B) Representative images of individual cell painting stains, target organelle and corresponding filter across GSC panel. Scale bars, 20 μm. IMX = ImageXpress high content screening platform. (C) Established high content screening, image and analysis workflow, followed by subsequent hit validation and exploration. Created in BioRender.

Previously, we have successfully adapted the cell painting protocol^30^^,^^38^ to explore drug MOA across cancer cell line panels from diverse lineages, including breast and esophageal adenocarcinoma.^39^^,^^40^^,^^41^ In the current study, we adapt the protocol to our panel of patient-derived GSCs (Figures 1A and 1B). It is our understanding that this study is among the first applications of phenotypic profiling using the cell painting assay to a panel of patient-derived GSC models. Image analysis, performed using a customized CellProfiler^30^^,^^38^^,^^42^^,^^43^ analysis pipeline, quantified up to 1,006 cellular features from each GSC. Principal-component analysis of these cellular features was carried out using the StratoMineR HCA platform (stratominer.com). Assay validation performed on nuclei count features confirmed assay reproducibility and signal-to-noise in 384-well plate format as suitable for high-throughput screening (HTS), based on industry-standard recommendations^44^ (Figures S1D and S3). Our phenotypic screening campaign performed across the panel of six GSC lines incorporated multiple approved and target-annotated small molecule compound libraries including a bespoke “Comprehensive anti-Cancer small Compound Library,” (C3L) designed to functionally evaluate known oncology targets across phenotypic screening assays^45^ (Table 1, materials and methods). The majority of these commercially available compounds (across all library sets) have been through phase I clinical trials. It is our view that this approach presents the potential for discovering novel drug repurposing and drug combination opportunities, in addition to identifying new therapeutic targets not previously explored in GBM.

**Table 1:** Screening libraries and concentrations used

Library	No. of compounds	Source	Concentrations used (nM)	Controls (nM)
TargetMol-Anti-Cancer	330	TargetMol #L2110 (2018)	10,000, 100, 10, 1	Paclitaxel (5 nM), Staurosporine (100 nM)
Kinase Chemogenomic Set (KCGS)	187	Structural Genomics Consortium https://cancertools.org/the-kinase-chemogenomic-set-kcgs/	1,000, 100	Paclitaxel (5 nM), Staurosporine (100 nM)
Prestwick FDA	1,280	Prestwick Chemical (∼2016) https://www.prestwickchemical.com/screening-libraries/prestwick-chemical-library/	10,000, 1,000	Staurosporine (1,000 nM)
LOPAC® (Library of Pharmacologically Active Compounds)	1,280	Merck/Sigma-Aldrich (LO4100)	3,000, 500	Staurosporine (1,000 nM)
Comprehensive Anti-Cancer Compound Set C3L	789	Custom (Athanasiadis et al., 2023^45^)	3,000, 300, 30, 3	Staurosporine (1,000 nM)

### High content cell painting screen, analysis and validation

**Figure 2:**
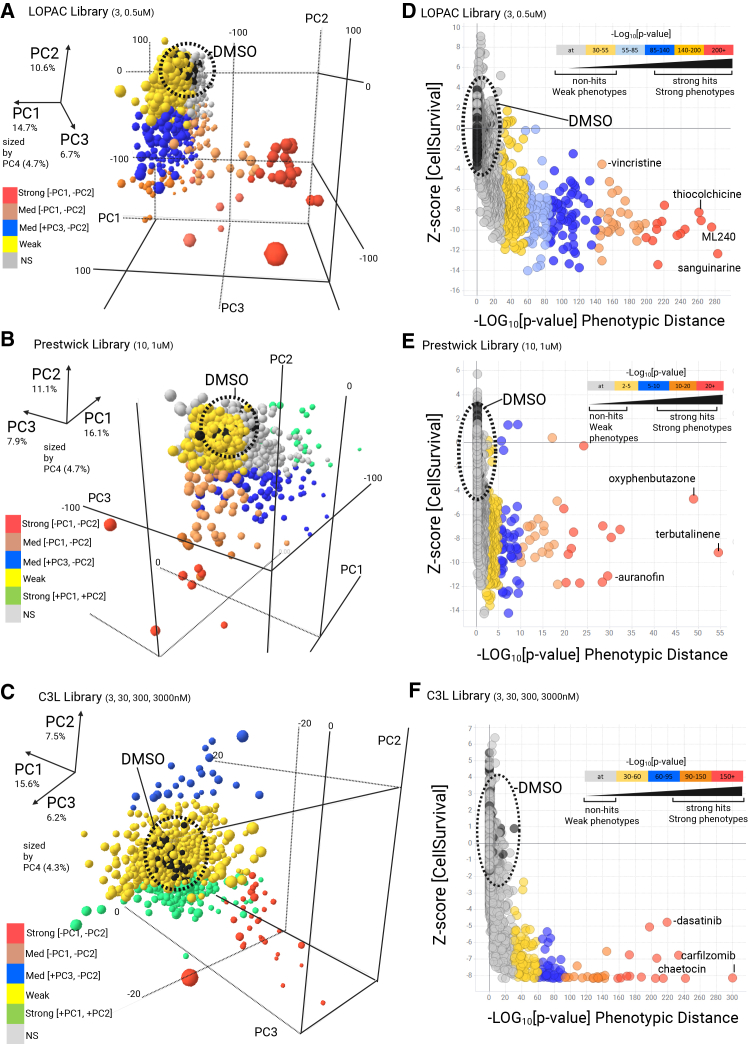
High-content image analysis/dimensionality reduction of drug screening data (A–C) Representative data of high content analysis across six GSC lines and three compound libraries (LOPAC, (A) Prestwick FDA (B) and C3L, (C) by 3D principal component analysis (PCA) (median aggregation to well level, *n* = 6 fields of view). DMSO controls circled in Black (*n* = 48 per plate). Magnitude of vector co-ordinates for PC1, 2, 3 (sized by PC4) are indicated by color, red-pink (strong –PC1, –PC2), blue (strong +PC3, –PC2), green (strong +PC1 +PC2), yellow/gray (weaker phenotypes). PC1-4 factor loadings are given (%). Factor loadings are provided in Table S7. (D–F) Phenotypic distance of screened compounds (LOPAC, D, Prestwick FDA, E and C3L, F). We employed 20 principal components (>60% explained variance) to compute a phenotypic distance (Euclidean) for each compound. The corresponding *p* values are plotted against cell survival (normalized *z* scores, *y* axis). Legend indicates strength of –log_10_[*p* value] distribution (five bins, red = maximal effect, reducing to gray where at = arbitrary threshold). Several example hits are labeled.

Our optimized GSC high content phenotypic screening and analysis workflow is outlined (Figure 1C) and described in detail in the material and methods section. Given the generally slow growth rates of the GSCs relative to standard cancer cell line cultures, compound exposure over 72 h during screening was selected to ensure at least one population doubling in all lines. The concentration of each cell painting reagent was optimized for the GCS panel and ImageXpress high content imaging platform (Table 2). In total 3,866 compounds were screened (varied doses), ∼2.2 million images collected (21 terabytes) representing a dataset of >62,000 datapoints. Image analysis was carried out via a custom CellProfiler pipeline and secondary multiparametric data analysis was performed using StratoMineR (Core Life Analytics)^46^ and TIBCO Spotfire software (Revvity), see STAR Methods. Each compound library was screened separately and representative principal component plots covering >60% of explained variance were created for LOPAC, Prestwick, and C3L libraries (Figures 2A–2C; Figure S4) to visualize distribution of active compounds from DMSO controls in phenotypic space. Further dimensionality reduction of these principal components was carried out, creating a phenotypic (Euclidean) distance for each compound, dose and cell line, relative to DMSO controls. We computed the phenotypic distance *p* values with the embedded R functions from StratomineR (see STAR Methods for more detail). The corresponding *p* value to this phenotypic distance was plotted against cell survival (*z* scores), based on DMSO normalized nuclei counts (Figures 2D–2F). Standard HTS quality control assessments were also carried out via nuclei count outputs from DMSO and staurosporine controls to evaluate assay signal-to-noise via *Z* prime robust (>0.35 minimum) across each plate (Table S2).

We examined the degree of clustering of compounds with a phenotypic response far from the DMSO controls, using a combination of *k*-means clustering and silhouette score analyses (Figures S5A and S5B).^47^ This analysis did not reveal a noticeable cluster structure and thus suggests a lack of morphological or phenotypic diversity of treatment effects across all screened libraries. Additionally, the distribution of the phenotypic distances across individual cell lines and libraries shows substantial heterogeneity in drug sensitivity, with particularly marked differences between the Prestwick and C3L libraries (Figure S5C).

Initial hit identification was based on two phenotypic criteria, a cell survival threshold (nuclei count *Z* score < –3) and phenotypic distance threshold (negative log_10_[*p* value] > 2) capturing changes in cell number and/or morphology following compound treatment in two or more GSC lines. Hit compounds were further triaged by de-prioritization of excessive examples of overtly cytotoxic agents (including common anti-cancer agents which have historically been investigated across pan-cancer indications, including GBM), such as microtubule disruptors, topoisomerase poisons and proteasome inhibitors. Compounds active at only high concentration (e.g., 3 μM), and/or with promiscuous or vague MOAs, or superseded by analogs against the same target, were also de-prioritized. Short-listed hits were taken forward for further investigation. Table S1 includes specified “priority group1/2” compounds versus “deprioritized” compounds, with added rationale.

**Figure 3:**
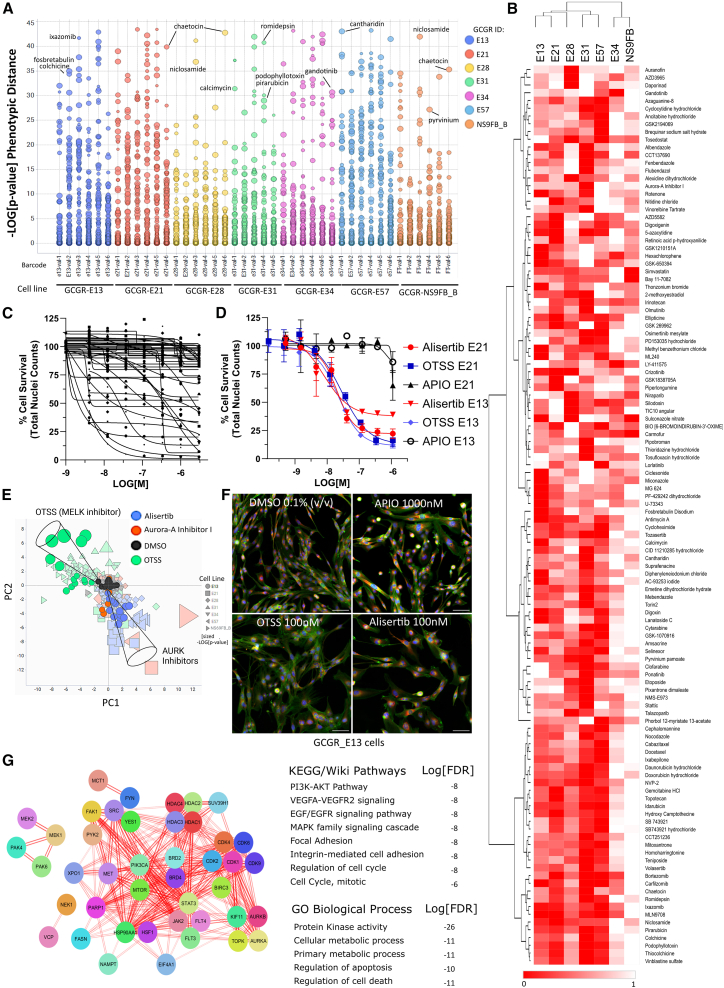
Hit validation from primary screening (A) Scatterplot representing phenotypic distance (negative –log_10_[*p* value]) across 211 compounds and six GSC lines, and one non-transformed neural stem cell line; colored by cell line, annotated by barcode and sized by dose (∼3 nM–1 μM). Example hits are labeled. (B) Representative heatmap of nAUC data from dose response data (normalized nuclei counts) across six GSC lines and a normal neural stem cell control line (NS69FB_B), scaled between 0 (dead) and 1 (100% viable). Potency (low AUC) is indicated in red (relative shading by row). Hierarchical clustering by Euclidean distance/complete linkage. (C) Representative normalized dose response data from a 384w plate (40 compounds per plate, seven point dose response [*n* = 1], DMSO *n* = 28) used to derive the nAUC. (D) Example of dose response validation of alisertib (AURK inhibitor, red) vs. OTSSP167 (MELK inhibitor, blue) and relatively inactive compound APIO-EE-07 (RKS/MSK2 inhibitor, black) across E13 and E21 cells. Data points are represented as mean ± stdev. (E) Example of distinct principal components/phenotypic space occupied by OTSSP167 (green) vs. alisertib (blue) and Aurora A kinase inhibitor (red) over multiple doses vs. DMSO (black). E13 cells are highlighted (circles) with vector direction indicated (black cones). All doses and replicates are shown, shaped by cell line and sized by –log[*p* value]. (F) Representative images (E13 cells) displaying differing phenotypes between alisertib, OTSSP167 and APIO-EE-07 (vs. DMSO) in spite of comparable IC_50_ curves. Staining, Hoechst (blue), phalloidin/WGA (green), endoplasmic reticulum (red). Scale bars, 100 μm. (G) STRING-Cytoscape network and enrichment analysis of validated hits, by annotated target(s) search (sources, PubChem, Selleck Chemicals, ChEMBL, and canSAR.ai). Lines indicate confidence of molecular interactions (edge score filter 0.4, text mining filter 0.06) with gene ontology (GO), KEGG, and Wiki terms associated the enrichment analysis, with log_10_[FDR] is given. The enrichment analysis is provided in Table S3.

Two hundred and eleven compounds (5.5% hit rate) were repurchased from different suppliers from that of the original screening libraries, for validation across GSC and a normal neuronal stem cell control line (NS69FB_B) by seven-point semi-log dose response (3–3,000 nM) phenotypic screening. Nuclei count data were also extracted from the phenotypic features and the validation set was analyzed via both multiparametric and univariate analysis on nuclei counts, with normalization to DMSO wells (Figures 3A–3C). The multiparametric analysis is presented as –log_10_[*p* value] phenotypic distance across all cell lines (Figure 3A) and we also converted dose response data to normalized area under the curve (nAUC, scaled between 0 and 1 with 100% death = 0) for validation ranking (nAUC <0.85 in any line) to indicate the potential of achieving a therapeutic window (Figure 3B). Examples of nAUC derived dose response data across each validation plate is shown (Figure 3C) with comparative dose responses between three example compounds: alisertib (Aurora A kinase inhibitor), OTSSP167 (MELK inhibitor), and APIO-EE-07 (RSK1/MSK2 inhibitor) (Figure 3D). In spite of similar dose response profiles between alisertib and OTSSP167, these compounds occupy distinct phenotypic space (Figure 3E and representative images; Figure 3F); hence, this approach allowed us to identify compounds with comparable cell survival, while inducing different phenotypic changes via distinct MOAs.

**Figure 4:**
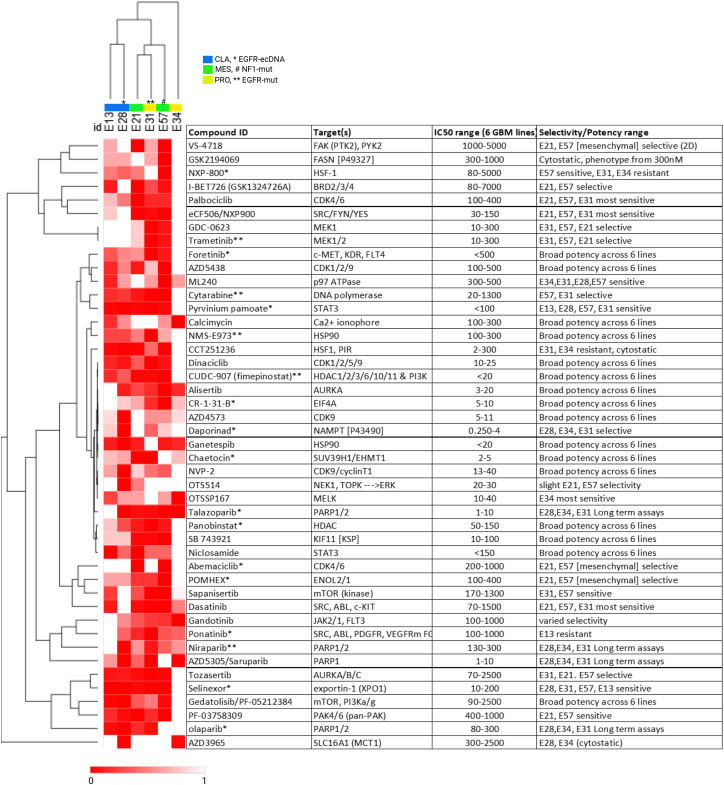
Validated compounds of primary interest Hierarchical clustering of IC_50_ heatmap (re-scaled 0–1 with low IC_50_ in red, high IC_50_ or “inactive” in white) alongside corresponding compounds name, annotated primary target(s), IC_50_ range (min to max) across six cell lines and indicated selectivity between lines (where applicable). ∗ Denotes predicted brain penetrant or ∗∗ known CNS/brain penetrance (40%; 18/45).

In this study, validated compound data lists were generated, including separate phenotypic distance and cell survival assay endpoint lists (Tables S1 and S2). One hundred and sixty four (164) compounds have been validated in dose response studies (Table S1), including 143 compounds originally identified among the 211 hits in the primary screen (68% validation rate) and 21 alternative compounds, selected against target classes represented in the hit list. As expected, there are clear indications of *in vitro* sensitivity of GSCs to topoisomerase poisons, anti-metabolites, proteasome and HDAC inhibitors, as well as particular sensitivity to microtubule targeting agents (taxanes, etc). A prioritized short list (45 compounds) was generated to explore more novel molecular targeted therapies which displayed activity upon GBM and selectivity across a heterogeneous GSC panel (Figures 3G and 4). A network enrichment analysis of this prioritized list was carried out, using the annotated primary protein target(s) for each compound, which demonstrates broad coverage of key target linked pathways, including PI3K-AKT, MAPK, and EGFR signaling (Figures 3G; Figure S6; Table S3). Agents of interest involve those targeting cell adhesion (focal adhesion kinase [FAK], SRC, YES, and FYN), transcription and translation (JAK2/STAT3, cyclin-dependant kinase 9 [CDK9], HDAC, and BRD), MAPK pathway/crosstalk (MEK1,2, PAK4, and 6), DNA damage/repair (TOPO and PARP), mitosis (AURK and KSP), metabolism (FASN and NAMPT) and protein folding/homeostasis (HSP90, HSF, and VCP/p97). The IC_50_ data of the prioritized 45 compounds from Figure 4 were combined with the RPPA and cytokine basal protein expression data (from Figure S2) across the GCGR cell line panel in order to create a similarity matrix which may suggest biomarkers for drug sensitivity or resistance (Figure S7; Table S4 [sheet1]).

For clarity, the similarity matrix was edited such that compounds appear on the x axis and protein names on the *y* axis (Figure S7B; Table S4 [sheet2]), with BLUE indicating negative correlations (which suggest protein expression drives sensitivity and are possible biomarkers for drug response) and RED indicating positive correlations (which suggest protein expression drives resistance and are possible targets for a drug combination). The original datasets used to create the similarity matrix are also provided (Table S4 [sheets 4 and 5]). As an example (Figure S7C), we observed fibronectin (FN1) expression negatively correlates with inhibition of FAK (VS4718, correlation −0.72), SRC (eCF506/NXP-900 [correlation −0.64], dasatinib [correlation −0.71], ponatinib [correlation −0.58]) as well as MEK inhibitors trametinib and GDC-0623 (correlation −0.58 each) and CDK4/6 inhibitors, palbociclib, and abemaciclib (correlations −0.67 and −0.41, respectively). Comparing the original potency data for FAK and SRC (IC_50_s) against FN1-normalized RPPA signal (FU) across the GCGR panel (Figure S7D), the negative correlation between FN1 expression and FAK and SRC inhibitor sensitivity appears consistent between high FN1 versus low FN1 expressing cells i.e., compare E21 versus E28 cells (Figure S7D, indicated arrows). Therefore, these data suggest high FN1 expression could act as a predictive biomarker for response to FAK or SRC inhibition, although further testing across a wider panel of GBM cell lines would be required to confirm this hypothesis.

In summary, there is clear chemical and target diversity within this validated hit list, beyond the expected highly potent cytotoxins, which provides a preliminary shortlist for rational drug-target selection and future systematic drug combination strategies with potential for personalized therapy.

### Secondary assays/exploration of target classes: Targeting transcriptional regulation

As it is beyond the scope of this article to follow up all of these hits in secondary assays, we have provided our full list of validated hit compounds for the GBM research community to explore and perform follow up investigation across similar and alternative GBM models. Within the scope of the current study, we have partially characterized two validated target classes (HDAC and CDK inhibitors) which demonstrated highly potent (low nanomolar) activity upon GSC survival (Table S1).

**Figure 5:**
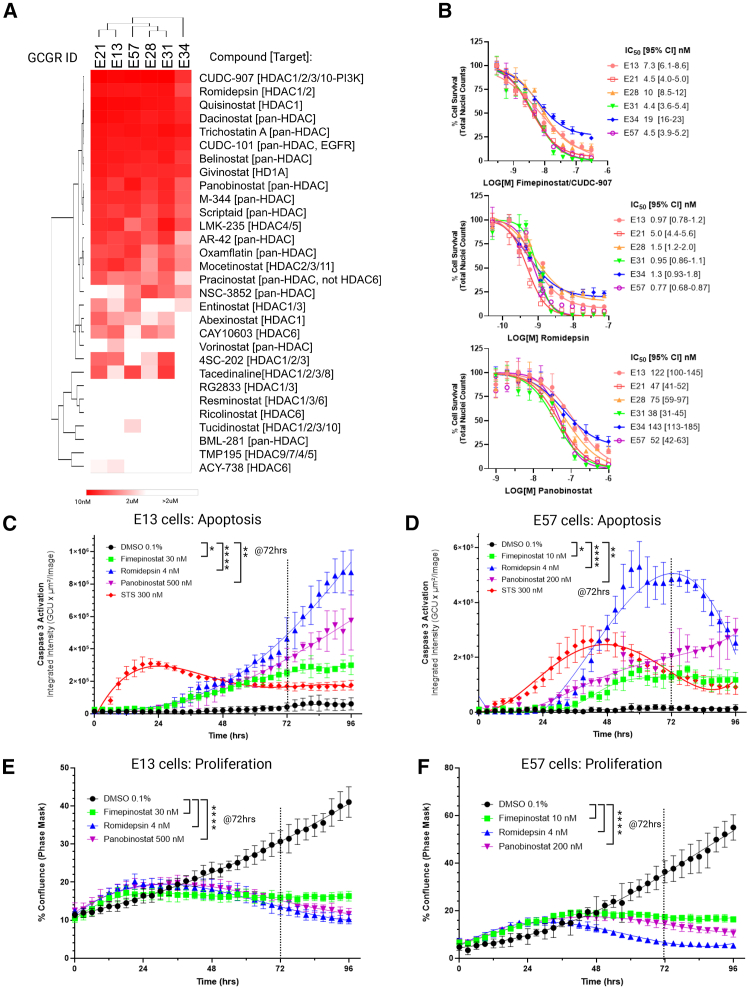
HDAC inhibitor library screening and profiling (A) Representative heatmap of IC_50_ values of HDAC inhibitors (8 point dose response, *n* = 6 fields of view per dose point) across six GSC lines, quantified by DMSO normalized nuclei counts at 72 h. The lowest IC_50_ compounds indicated in red (from 10 nM to 2 μM, inactive >2 μM indicated in white). Hierarchical clustering by complete linkage, Euclidean distance using Morpheus (https://software.broadinstitute.org/morpheus). (B) Dose response validation of fimepinostat (CUDC-907), romidepsin and panobinostat with indicated IC_50_ values (nanomolar, nM) and IC_50_ 95% Confidence Interval range (95% CI, 72 h, *n* = 3, data points are represented as mean ± stdev). (C and D) Quantification of apoptosis by live cell imaging over time on (E13 cells C and E57 cells D, imaged every 3 h for 96 h, *n* = 3, data points are represented as mean ± stdev) at indicated IC_80_ values with staurosporine control (STS, 300 nM, red, *n* = 6). Significance of induced apoptosis, relative to DMSO controls (*n* = 6), at 72 h (dotted line) is indicated (One-way ANOVA, corrected *p* values, E13 cells, ∗*p* = 0.0172, ∗∗∗*p* = 0.003, ∗∗*p* = 0.0027; E57 cells, ∗ 0.0428, ∗∗∗∗*p* < 0.0001, ∗∗*p* = 0.0025, *n* = 3). (E and F) Quantification of proliferation over time (imaged at 3-h intervals for 96 h), at indicated IC_80_ values (E13 cells, E; E57 cells, F). DMSO control (0.1% v/v, *n* = 6) is represented in black versus fimepinostat (30 nM, *n* = 3, green), romidepsin (4 nM, *n* = 3, blue), panobinostat (500 nM, *n* = 3, purple). Data points are represented as mean ± stdev. Significance of anti-proliferation effects, relative to DMSO controls (*n* = 6), at 72 h (dotted line) is indicated (One-way ANOVA, corrected *p* values, ∗∗∗∗*p* < 0.0001).

Targeting transcriptional regulation (1), HDAC inhibitors: we further explored a focused library of 54 structurally distinct HDAC inhibitors by dose-response, as our screening data demonstrated the significant potency of this class of compounds across our GSC panel (e.g., romidepsin, panobinostat, etc.) (Figure 5). Commercially available HDAC inhibitors were purchased and 384-well dose response plates were prepared and screened against the same six GSC line panel used for primary screening. Compounds were ranked by potency using the cell survival endpoint (normalized nuclei counts) (Figure 5A). Unsurprisingly, the most potent compounds were pan-HDAC or HDAC1/2 inhibitors, although the top ranked compound (with IC_50_ values below 20 nM across all cell lines) was the dual HDAC/PI3K inhibitor, fimepinostat (CUDC-907) which is currently undergoing phase II clinical trials in pediatric brain tumors (NCT03893487). Fimepinostat, romidepsin and panobinostat were further validated by performing dose-response and cell cycle analysis (Figures 5B; Figures S8A–S8C). Proliferation/cell survival over 72 h is non-selectively inhibited across all cell lines, with cell cycle effects generally being limited to either small G_0_/G_1_ effects or strong G_2_/M arrest (Figures S8A–S8C). Live cell imaging on E13 and E57 cells over 96 h, with an intracellular caspase activity reagent showed a gradual but continuous induction of apoptosis from 24 h (Figures 5C and 5D) with corresponding loss of proliferation from 24 to 36 h (Figures 5E and 5F). Staurosporine (STS, 300 nM) was used as a positive control for the apoptotic signal. On target HDAC inhibitor activity was confirmed by western blot (Figure S9).

**Figure 6:**
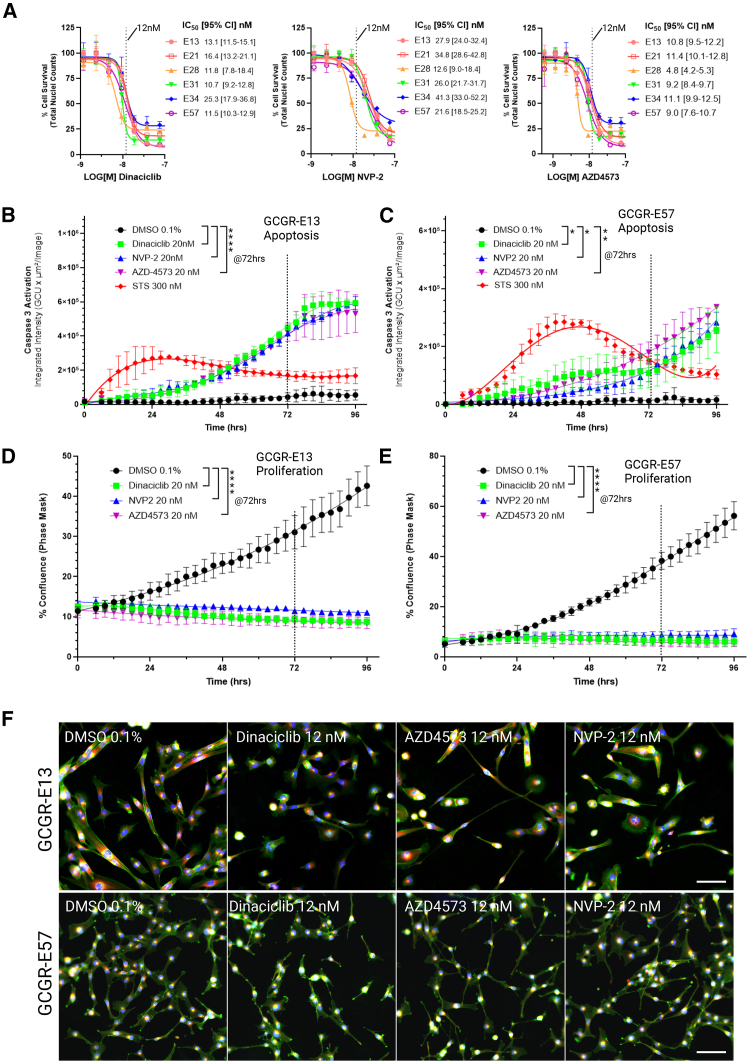
Targeting CDK9 in GBM (A) Dose response validation and IC_50_ values with IC_50_ 95% confidence interval range (95% CI) of Dinaciclib, NVP-2 and AZD4573 (72 h, *n* = 3, data points are represented as mean ± stdev). Twelve nanomolar (12 nM) concentrations are indicated with a dotted line. (B and C) Quantification of apoptosis by live cell imaging over time (E13 cells, B; E57 cells, C, imaged at 3 h intervals for 96 h, *n* = 3, data points are represented as mean ± stdev), all compounds at 20 nM concentrations with staurosporine control (STS, 300 nM, *n* = 6, red). Significance of induced apoptosis, relative to DMSO controls (*n* = 6), at 72 h (dotted line) is indicated (One-way ANOVA, corrected *p* values, E13 cells, ∗∗∗∗*p*=<0.0001, *n* = 3; E57 cells, ∗*p* = 0.024–0.026, ∗∗*p* = 0.0010, *n* = 3). (D and E) Quantification of proliferation over time (E13 cells, D; E57 cells, E). DMSO control (0.1% v/v, *n* = 6) is represented in black, versus dinaciclib (green, *n* = 3), NVP-2 (blue, *n* = 3), AZD4573 (purple, *n* = 3). Significance of anti-proliferative effects at 72 h (dotted line) is indicated (One-way ANOVA, corrected *p* values, ∗∗∗∗*p*=<0.0001). (F) Representative images of dinaciclib, AZD4573 and NVP-2 at ∼ IC_50_ value (12 nM) vs. DMSO on E57 and E13 cells. Staining, Hoechst (blue), phalloidin/WGA (green), endoplasmic reticulum (red). Scale Bars, 100 μm.

Targeting transcriptional regulation (2), CDK9 inhibitors: CDK inhibitors have been extensively investigated for their anti-cancer activity^48^^,^^49^ and several CDK inhibitors feature in our validated hit list, including approved CDK4/6 inhibitors abemaciclib and palbociclib. In particular, the CDK1/2/5/9 inhibitor, dinaciclib, as well as the selective CDK9 inhibitor, NVP-2,^50^ both show potent activity across multiple GSC lines (11–40 nM IC_50_ across the six GSC lines) (Figure 6A). In addition, a more potent, second generation CDK9 inhibitor, AZD4573, was included in this validation stage and also demonstrated low nM (7–27 nM) activity across the six GSC lines (Figure 6A).

Dinaciclib is a multi-CDK inhibitor which has gained clinical interest as it targets both cell cycle progression (via CDK1/2) and transcription (via CDK9), with this “dual targeting” mode shown to be effective when combined with cisplatin in ovarian and endometrial cancers.^51^^,^^52^ Validation of dinaciclib, NVP-2 and AZD4573 across our GSC panel indicates broad potency at 72 h across all the cell lines tested, with G_2_/M arrest being initiated at concentrations in excess of 20 nM i.e., above the IC_50_ values observed (Figures S8D–S8F). Live cell imaging of these compounds, on E13 and E57 cells over 96 h and in the presence of intracellular caspase activity reagent, showed rapid induction of apoptosis beginning at <24 h from compound addition (Figures 6B and 6C) with corresponding immediate inhibition of proliferation, as observed from % confluence quantification (Figures 6D and 6E). Representative images of the phenotypic effects of these compounds at their IC_50_ concentration are shown (Figure 6F). This suggests transcriptional dysregulation via CDK9 inhibition has a significant effect upon cell survival, independent of CDK1/2 inhibition. Overall, AZD4573 has improved cellular potency compared to dinaciclib and NVP-2 (Figures 6A–6F, compare observable 12 nM effects vs. DMSO).

**Figure 7:**
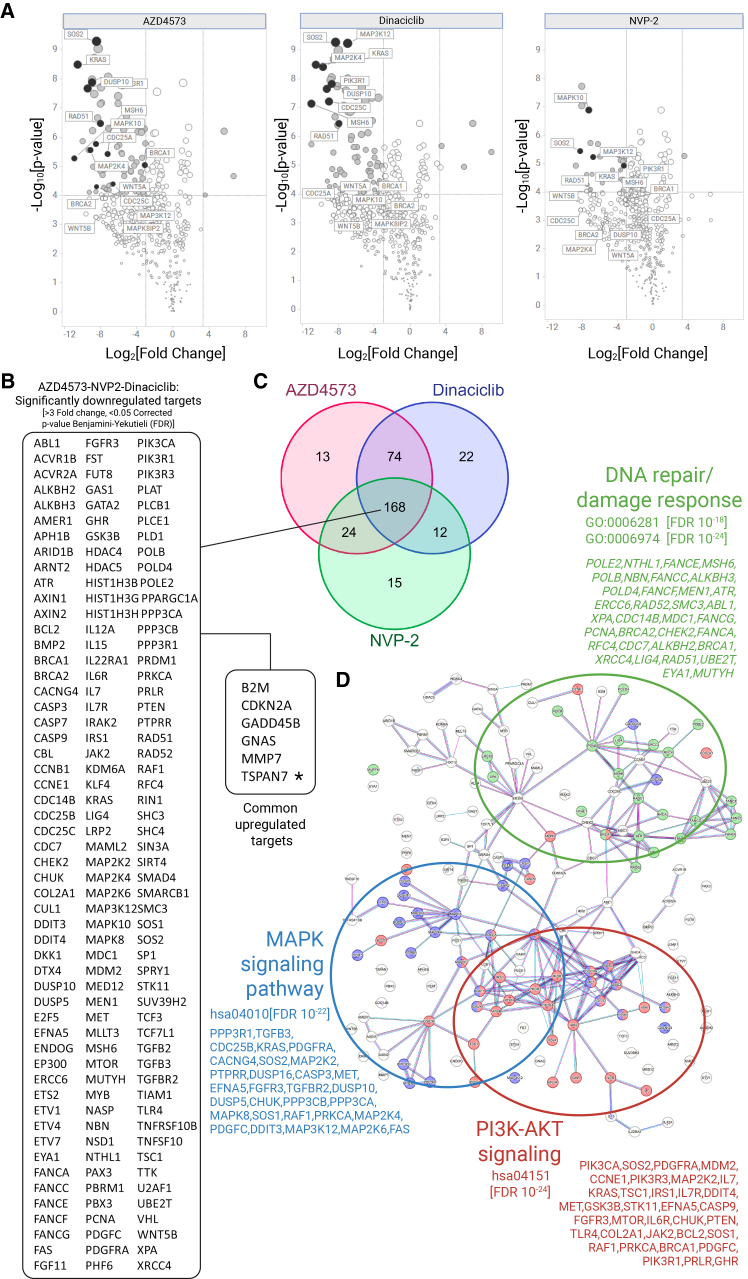
Differential gene expression (DE) analysis of three CDK9 inhibitors on E13 cells from NanoString transcriptomics (A) Volcano plots of –log_10_[*p* value] vs. log_2_[fold change] from the differential analysis of E13 cells treated with AZD4573, dinaciclib, and NVP-2 (20 nM, *n* = 3, 24 h), relative to DMSO control (0.1% [w/w], *n* = 3). Significant fold changes are indicated (shaded gray circles) with select genes labeled (black circles). (B) Top 168 genes dysregulated by all three compounds (>3-fold change, *p*= <0.05 [false discovery rate controlled by Benjamini-Yekutieli corrected *p* values]). Large section, significantly downregulated genes; small section, upregulated genes. TSPAN7 is annotated with ∗. (C) Venn diagram comparing gene hits across all three compounds. (D) Network enrichment analysis (STRING) of top 168 genes. Enriched genes involved in DNA damage and repair (green), MAPK signaling (blue) and PI3K-AKT pathway (red) are highlighted. GO and KEGG terms with FDR is included. Enrichment tables and further data are provided (Table S5; Figures S10–S12).

From these studies it is clear that targeting HDAC and CDK9, and hence transcriptional regulation, results in a cytotoxic benefit against GSCs, beyond the effects of prolonged cell-cycle arrest (with the exception of the highly potent molecule, romidepsin). To further explore the MOA of the CDK9 inhibitors on GSC lines, we performed NanoString transcriptomic analysis on E13 cells that were exposed to 20 nM of dinaciclib, AZD4573, or NVP-2, alongside DMSO controls, for 24 h following compound addition. After quality control filtration and removal of housekeeping/control gene sets (post-normalization), 467 genes were available for the differential expression and network pathway analysis, between untreated and treated cells. Representative Volcano plots were generated for each compound with some highly significant genes labeled (Figure 7A).

Not surprisingly, a large number of genes (168) were significantly dysregulated across all three compounds (Figure 7B), visualized by Venn diagram (Figure 7C). STRING network enrichment analysis of these 168 genes (Figure 7D) showed significant enrichment of processes downregulating the MAPK cascade (e.g., *KRAS*, *MAP2K4*, *MAPK8*, *MAP2K6*, and *DUSP10*), PI3K-AKT signaling (e.g., *PIK3CA/CB/CD* [p100α,β,δ], *IRS1*, *FGFR3*, *mTOR*, and *MYB*), DNA damage response/repair (e.g., *RAD51*, *RAD52*, *BRCA2*, *PCNA*, and *POLE2*) and apoptotic signaling (Figure 7D). The complete network analysis for each compound is provided (Figures S10–S12) with enrichment tables and additional data (Table S5). MCL-1, an anti-apoptotic BCL2 family member, is a reported CDK9 transcriptional and protein target.^53^ This probe is not present in the NanoString panel; however, the functional activity of these CDK9 inhibitors was confirmed by western blot across E13 and E57 cells (Figure S13). RNA polymerase II (RPB1/RNAP2) and MCL1 protein was significantly reduced (relative to DMSO controls) after treatment with dinaciclib, NVP-2, and AZD4573 (100 nM) (Figures S13A and S13I). Inhibition of PI3K-AKT signaling was observed via reduction in AKT phosphorylation (pSerine-473, Figure S13K) and corresponding increase in ERK1/2 phosphorylation via a negative feedback loop^54^^,^^55^^,^^56^^,^^57^ (Figure S13D and S13E). Phospho-p38 MAPK activity was modestly decreased in E57 cells upon treatment, although expression of total p38 protein was more clearly reduced in both cell lines following treatment, compared to DMSO controls (Figures S13B and S13C). RAD51 expression was weakly decreased upon CDK9 inhibition and the significant upregulation of TSPAN7, observed at the mRNA level, was not translated at the protein level at 24 h post-treatment (Figure S13G).

Finally, we investigated potential synergistic drug combinations with some of our most potent hits which are currently clinically approved drugs: specifically, dinaciclib against romidepsin (pan-HDAC inhibitor), idarubicin (TOPOII inhibitor), and ixazomib (proteasome inhibitor) (Figures S14–S16). Querying the Cancer Dependency Map database^58^ (depmap.org/portal), a strong “cell death” correlation was observed between dinaciclib and romidepsin/idarubicin/ixazomib (Figures S14–S16), (R^2^ = 0.62 to 0.67) across 564 human cancer cell lines (PRISM Repurposing Public 24Q2 dataset). In order to establish if this correlation could predict synergistic interactions, we carried out multiple drug combinations (7 × 7 dose response matrices) across E13 and E57 cells with quantification from nuclei staining/counts (72 h post-exposure). While the drug combination of dinaciclib with either romidepsin, idarubicin, and ixazomib indicates highly potent additive effects upon GSC survival, synergistic activity was not observed in this instance (δ-surface plots, Figures S14–S16).

## Discussion

The clinical treatment of GBM has not significantly improved median patient survival in more than 20 years, with advances in the genomic characterization of the disease only highlighting the complexity of the clinical challenge. To address this challenge, a number of phenotypic screens of drug libraries and drug combinations performed in complex *ex vivo* or *in vitro* GBM models have recently been reported.^59^^,^^60^ However, screens reported to date have been limited in terms of the number of compounds, phenotypic endpoints and omission of defined tissue culture conditions which preserve GSC characteristics. In this study, our aim was to attempt to address the unmet clinical need in GBM by applying a comprehensive target agnostic, high content phenotypic screen, of thousands of compounds across a well characterized panel of patient-derived GSC models. The drug libraries were selected to support repurposing of existing oncology drug discovery programs and included small molecules and drugs with high target selectivity across known oncology targets which have passed Phase I or have been FDA/EMA approved. We have chosen to not exclusively use blood-brain barrier (BBB)/CNS penetrant drug libraries in order to provide a comprehensive and unbiased evaluation of distinct drug and target classes upon GSC phenotypes—that said, 45% of our validated compounds (74/164) are computationally predicted to be brain penetrant^61^ (Table S1). It is our hope that this will be informative toward future medicinal chemistry efforts in generating CNS penetrating small molecules against such targets, or possible incorporation of validated hit compounds into emerging BBB penetrating nanoparticles, designed to effectively deliver drugs and drug combinations to GBM. In total 3,866 compounds were screened over multiple concentrations, in order to capture the most potent dose-dependent cellular phenotypes beyond simple viability measurements. Our GBM models comprised six heterogeneous, patient-derived GSC lines, representing three of the major transcriptomic subtypes (CLA, MES, and PRO). We applied the multiparametric high content “cell painting” assay to screen five drug libraries across the six GSC lines, and provide a validated list of 164 biologically active compounds on human GSC survival and phenotypic activity (Figure 4; Tables S1 and S2).

Common cytotoxic agents, such as microtubule targeting agents, topoisomerase poisons, proteasome and HDAC inhibitors, are prevalent in our hit list and many have previously been investigated in GBM (and other cancers). Second generation taxanes, such as cabazitaxel, have shown modest effects with respect to mOS in phase I/II clinical trials (NCT01866449).^62^ Abeotaxane (TPI-287), a brain penetrant taxane, showed some promise in phase I trials when combined with bevacizumab (NCT01933815), although confirmation of efficacy via phase II trials is required.^63^ A recent meta-analysis of the use of the topoisomerase II inhibitor etoposide (44,850 patients over 624 studies published from 1976 to 2011) showed improved mOS (15.66 months vs. 13.27, *p* = 0.026) over topoisomerase I inhibitor, irinotecan. Results are pending for a novel brain penetrant topoisomerase II inhibitor, berubicin (WP744), which has been granted fast track designation by the US FDA.^64^ Proteasome inhibitors have been clinically useful in treating blood cancers, such as myeloma,^65^ unfortunately a phase III study of the brain penetrant proteasome inhibitor, marizomib, did not improve mOS against unstratified patients.^66^ The HDAC inhibitor class, such as romidepsin, in general has shown considerable promise as single agents for GBM treatment in the pre-clinical setting, but without translational success.^67^ Clinical trials investigating their use in drug combination therapies, for example combining panobinostat or vorinostat with radiation, bevacizumab and/or temozolomide, have not proved particularly effective.^68^^,^^69^^,^^70^ Arguably, this lack of clinical success could be attributed to trial design, limited predictive biomarkers and inadequate assessment of brain penetration.^71^^,^^72^ Many of these agents have a narrow therapeutic window and significant systemic toxicities limiting their effectiveness. Nevertheless, novel drug delivery technology, such as antibody-cytotoxin conjugates, brain tumor-specific nanoparticles^73^^,^^74^^,^^75^ and intra-cranial drug delivery,^76^ could still render these potent molecules clinically useful in the future.

Other molecular targeted compounds identified from our screen (including those targeting cellular adhesion, transcriptional regulation, DNA damage/repair, cell cycle/mitosis/proliferation, metabolism, and protein folding/homeostasis) provide a basis for rational drug combination hypotheses and systematic co-inhibition strategies. For example, FAK and SRC family kinases (SFKs) are downstream effectors of the integrin family of extracellular matrix receptors and FAK is believed to influence GBM pathogenesis and brain tissue invasion.^77^^,^^78^^,^^79^ SFKs are recognized drug targets in GBM^80^^,^^81^^,^^82^ and PYK2 (FAK2),^83^ integrin-linked kinase^84^ and SRC all show high basal expression in our GSC models (Figure S2). Synergistic drug combinations of FAK or SRC inhibitors with MEK inhibitors have been described in other cancers^85^^,^^86^ and we report the combination of VS-4718 (FAK inhibitor) with trametinib (MEK1/2 inhibitor) to be quite effective in heterogeneous GBM *in vivo* models.^87^ In fact, recently the combination of the dual RAF/MEK inhibitor Avutometinib with FAK inhibition has been shown to be effective against pre-clinical models of melanoma brain metastases.^88^ Moreover, a phase I/II clinical trial combining avutometinib and defactinib (VS-6063) for NF1^del^ or BRAF^mut^ brain tumors has been initiated (5G-RUBY trial, NCT06630260) while the US FDA has recently granted accelerated approval for the avutometinib/defactinib combination for KRAS^mut^, low grade serous ovarian cancer (RAMP-201, NCT04625270, May 2025). Furthermore, a similarity matrix created from basal protein expression data (RPPA, cytokine arrays, 79 probes, Figure S2) and IC_50_ data (45 compounds, Figure 4) across the GCGR panel, suggests high fibronectin (FN1) expression could act as a biomarker for FAK/SRC and/or MEK inhibitor response (Figure S7; Table S4). FN1 has been reported as a potential prognostic marker in several cancers,^89^^,^^90^^,^^91^ as well as driving drug resistance.^92^^,^^93^^,^^94^ It has been shown in cervical cancer cells that high FN1 expression drives FAK activity, and subsequent FAK inhibition abrogates pro-survival and invasive signals.^95^ This negative correlation between high FN1 expression and FAK/MEK inhibitor response is potentially useful as a predictive biomarker; however, further work will be required to establish the clinical utility of this observation.

Exploiting GSC vulnerabilities with respect to cellular adhesion and combining with other druggable routes (e.g., EGFR/MAPK/PI3K signaling) remains a viable strategy for GBM treatment. Additionally, we have identified distinct sensitivities to molecules targeting heat shock factor-1 (HSF1; CCT251236), EIF4A (CR-1-31-B), SUV39H1 (chaetocin), FASN (GSK2194069), NAMPT (daporinad), enolase-2 (POM-HEX), exportin-1 (selinexor), and JAK2 (gandotinib, pacritinib) (Figure 4). Furthermore, our examination of the basal expression of our GSC lines by reverse phase protein and cytokine array also reveals actionable targets via high expression of forkhead box A1 (FOXA1) and interleukin-8 (IL-8) (Figure S2). FOXA1 is usually associated with hormone dependent cancers and can drive endocrine resistance in estrogen-positive breast cancer,^96^ or MAPK activation in prostate cancer,^97^ in an IL-8 dependent manner. Targeting IL-8 via humanized anti-IL8 antibody^37^ is potentially another combination strategy that could be systematically investigated alongside our prioritized hits (Figure 4; Table S1).

In this study we followed up two drug target classes, HDAC and CDK inhibitors, which displayed low nanomolar activity upon inhibiting cell survival across the heterogeneous panel of GSCs. To further explore the HDAC inhibitor class we screened a collection of 54 HDAC inhibitors. Fimepinostat (CUDC-907) was outstanding as the most potent molecule, with <20 nM IC_50_ values across the GSC panel. Fimepinostat, a dual PI3K/HDAC inhibitor, is currently undergoing clinical trials for pediatric/young adult brain cancers/solid tumors (NCT03893487), and is potentially a candidate for targeted drug delivery and drug combination strategies. We compared fimepinostat with structurally distinct HDAC inhibitors, romidepsin and panobinostat. While there was a varied cell cycle response between cell lines (Figure S8), there was a common intra-cell line response between molecules, suggesting activity is most likely mediated through the anti-HDAC activity of fimepinostat rather than its anti-PI3K activity. Indeed, comparison between fimepinostat and potent, selective PI3K inhibitors (all tested at 30 nM) demonstrate substantially weaker activity of PI3K inhibition upon GSC proliferation, with only dual PI3K/mTOR inhibitor, gedatolisib (PF-05212384), showing any significant effect (Figure S17). While the lack of success in targeting GBM with PI3K monotherapy may indicate a lack of true oncogenic addiction to PI3K signaling, drug combinations with PI3K/mTOR/AKT inhibitors are still viable options against PTEN deficient, GBM tumors.^98^

Targeting transcriptional regulation has previously been shown to be effective against cancer stem cells (including brain cancers);^99^^,^^100^^,^^101^ hence, we also characterized potent CDK9 inhibitors dinaciclib, NVP-2 (both screening hits) and a more recent selective CDK9 inhibitor, AZD4573. CDK9 is an atypical CDK in that it is a key regulator of RNA polymerase II (RPB1/RNAP2) transcription initiation, elongation and termination, playing a critical role in transcriptional regulation.^102^ Dinaciclib has near equal potency against CDK1/2 (and 5), whereas NVP-2 and AZD4573 are classed as CDK9 selective.^50^^,^^103^ AZD4573 has progressed to clinical trials (NCT04630756) and is well tolerated, although CNS penetrance and solid tumor efficacy remains to be determined. Radiosensitization of cancer cells with CDK9 inhibitors and drug combinations with DNA damaging agents have previously been reported,^104^^,^^105^^,^^106^ including opportunities in GBM.^107^ In our *in vitro* assays, dinaciclib, NVP-2 and AZD4573 are highly potent across all GSC lines tested, being strongly antiproliferative with predominant G_2_/M arrest and induction of apoptosis. Differential gene expression analysis of GSC cells exposed to CDK9 inhibitors, reveals highly significant decreases in the expression of genes regulating the cell cycle, DNA damage and repair, anti-apoptosis, PI3K, and MAPK signaling (Figure 7; Figures S10–S12; Table S5). Western blotting confirmed the suppression of RNA polymerase II, anti-apoptotic protein MCL1, phospho-AKT^Ser473^, and total p38 MAPK (Figure S13) by all three compounds. These results indicate that the CDK inhibitors are reprogramming the GSCs into a more vulnerable state, leading to inhibition of cell survival and, together with previous publications implicating CDK9 as a therapeutic target in GBM,^107^ suggest targeting CDK9 is worth further exploration in other GBM models and with other drug combinations.

Overall, our screening results presented here serve as a foundation for a systematic approach to the identification, validation and prioritization of novel therapeutic strategies in GBM including future drug combinations, that could be used concurrently or following standard treatment protocols for GBM. Importantly, we have used patient-derived GSC models, grown under stem-like conditions on a laminin rich ECM scaffold to prioritize follow up investigations of highly potent small molecules active across a genetically distinct and heterogeneous GSC panel. The objective of our phenotypic screening assay was to identify multiple drug targets and mechanisms-of-action which target heterogeneous GSC phenotypes and thus complements the existing focus on known oncogenic drivers that represent established drug target classes. We anticipate that this approach will identify new therapeutic targets and drug combination hypotheses that target GSC phenotypes rather than individual pathways and thus may be more resilient across heterogeneous patient populations and adaptive drug resistance mechanisms.

In conclusion, our high content screening followed by multi-cell line validation across primary and secondary phenotypic assays provides the largest unbiased survey of small molecule therapeutic classes and oncology targets upon GSC phenotypes that we are aware of. We provide our full list of validated hits with their primary target-assignments (Table S1) which are active upon GSC morphology and cell survival for the research community to explore further. We encourage additional validation across more complex animal and novel alternative (non-animal) models of GBM to further determine their translational utility. Future studies may also include expanded screening across larger target-annotated and diverse chemical libraries to identify novel chemical starting points and novel targets for new GBM drug discovery programs. In addition, further profiling of phenotypic screening hits across a larger number of GSCs lines and integration of phenotypic with molecular data, in order to identify synthetic lethality relationships and/or predictive biomarkers of response, will support additional personalized medicine strategies for both existing and new drug target classes in GBM. Importantly this unbiased approach can identify new therapeutic opportunities for the majority of GBM patients which do not fall into well-defined subtypes characterized by well-known druggable driver genes, thus expanding the scope and inclusion criteria of personalized medicine strategies in GBM.

### Limitations of the study

While all of the GSC lines used in our study have been subjected to deep molecular profiling at transcriptome, epigenome and genome-wide level to confirm expression of all of the molecular hallmarks of GSCs and show consistent tumor initiation capacity *in vivo* (personal communication with the Cancer Research UK Glioma Cellular Genetics Resource); to formally confirm that our compound hits specifically target the cancer stem cell properties of these cells, functional stem cell assays, such as *in vitro* extreme limiting dilution assays or xenotransplantation limiting dilution assays, would be required.

The MOA studies of the CDK9 inhibitors using the NanoString transcriptomic platform are restricted to canonical cancer signaling, cell growth, and survival pathways. Further in-depth profiling of selected drug responses using single-cell(sc)RNA-Seq technologies will very likely reveal further insights into drug MOA in GSCs including changes in glioma stem cell state subtypes.

Our phenotypic screening study restricted to 6 cell lines representing the three bulk transcriptomic subtypes (MES, CLA, and PRO) is an oversimplification of GBM heterogeneity and further screening and/or hit validation across a larger panel of molecularly annotated GSC lines incorporating scRNA-Seq technologies where feasible will reveal further insights upon the impact of drug treatment on the heterogeneity and plasticity of GSC subtypes.

While our multiparametric high content phenotypic analysis revealed many compounds that alter the morphology of the GSCs we did not identify many discrete morphological clusters. This may be an inherent property of limited GSC shape; however, performing the cell painting assay at earlier time points may overcome confounding effects of cell death upon cell morphology and potentially capture additional viability-independent phenotypes.

## Resource availability

### Lead contact

Further information and requests for resources and reagents should be directed to and will be fulfilled by the lead contact, Neil O Carragher (n.carragher@ed.ac.uk).

### Materials availability

This study did not generate new unique reagents.

### Data and code availability

Data repository for access to unprocessed image analysis datasets, drug library plate-maps and annotations, related information and R scripts are provided: https://datashare.ed.ac.uk/handle/10283/9113 (see key resources table).

## Acknowledgments

This work was funded by a joint 10.13039/501100000289Cancer Research UK (C42454/A28596) and 10.13039/501100002203The Brain Tumour Charity award (GN-000676) to D.E., N.O.C., and M.C.F. and a 10.13039/501100000833Rosetrees interdisciplinary award (ID2022\100030) to D.A.O and N.O.C. The Glioma Cellular Genetics Resource (gcgr@ed.ac.uk) was supported by the 10.13039/501100000289Cancer Research UK (10.13039/501100000289CRUK) Center Accelerator Award (A21922) to S.M.P. We wish to thank David Egan and Victor Wong for advice and assistance in using StratoMineR software for multiparametric high content analysis. We also acknowledge the 10.13039/501100000030Institute of Genetics and Cancer Host and Tumor Profiling Unit microarray services facility for supporting forward and reverse phase protein array studies.

## Author contributions

R.J.R.E contributed to the project administration, supervision, investigation, formal analysis, visualization and validation of the data as well as drafting the original manuscript, and writing – reviewing and editing. P.W.K.N. contributed to investigation, formal analysis, and writing – reviewing and editing. M.F. contributed to investigation, formal analysis, and writing – reviewing and editing. J.C.D. contributed to project administration, supervision, investigation, and writing – reviewing and editing. V.S.-B. contributed to formal analysis, visualization and validation of the data, and writing – reviewing and editing. D.A.O. contributed to investigation, visualization and validation of the data, and writing – reviewing and editing. A.M. contributed to investigation and formal analysis. A.F.M. contributed to the investigation and formal analysis as well as writing – reviewing and editing. C.D. contributed to investigation and formal analysis. G.M.M. contributed resources, data, and writing – reviewing and editing. S.M.P. contributed resources, data, and writing – reviewing and editing. M.M. contributed to investigation and formal analysis. D.E. contributed resources and writing – reviewing and editing. V.B. contributed to supervision, investigation, and writing – reviewing and editing. M.C.F. contributed to conceptualization, funding acquisition, supervision, investigation, and writing – reviewing and editing. N.O.C. contributed to the conceptualization, funding acquisition, project administration, supervision, investigation, formal analysis as well as drafting the original manuscript, and writing – reviewing and editing.

## Declaration of interests

S.P. is a co-founder, shareholder and Chief Scientific Officer of Trogenix Ltd.

N.O.C. is a co-founder, shareholder and management consultant for PhenoTherapeutics Ltd.

N.O.C and M.C.F. have held advisory positions and are shareholders in Amplia Therapeutics Ltd.

N.O.C. had patents pertaining to discovery of the SRC/YES1 inhibitor, eCF506/NXP900 (EP3298015B1, JP6684831B2, US10294227B2, CN107849050B, and CA3021550A1) licensed to Nuvectis Pharma and has received grant funding from Nuvectis Pharma.

## STAR★Methods

### Key resources table

**Table:** 

REAGENT or RESOURCE	SOURCE	IDENTIFIER
**Antibodies**		

Rabbit monoclonal anti-MCL1 (D35A5)	Cell Signaling Technology	Cat# 5453
Rabbit monoclonal anti-BCL2 (D5568)	Cell Signaling Technology	Cat# 4223
Mouse monoclonal anti-RNA polymerase II RPB1 clone H5	Biolegend, Inc.	Cat# 920203
Rabbit polyclonal anti-TSPAN7	Life Technologies (Invitrogen division)	Cat# 18695-1-AP
Rabbit monoclonal anti-acetyl Histone H3 (Lys9) (C5B11)	Cell Signaling Technology	Cat# 9649S
Mouse monoclonal anti-Histone H3.1/3.2 (1D4F2)	Active Motif	Cat# 61630
Rabbit monoclonal anti-phospho AKT (Ser473)	Cell Signaling Technology	Cat# 4060S
Rabbit polyclonal anti-AKT	Cell Signaling Technology	Cat# 9272S
Rabbit monoclonal anti-phospho-p44/42 MAPK (ERK1/2) (Thr202/Tyr204) (D13.14.4E)	Cell Signaling Technology	Cat# 4370S
Rabbit polyclonal anti-phospho-p38 MAPK (Thr180/Tyr182)	Cell Signaling Technology	Cat# 9211
Rabbit polyclonal anti-p38 MAPK	Cell Signaling Technology	Cat# 9212
Rabbit polyclonal anti-phospho-*c*-Jun (Ser73)	Cell Signaling Technology	Cat# 9164
Mouse monoclonal anti-alpha-Tubulin (DM1A)	Cell Signaling Technology	Cat# 12351
Anti-Rabbit IgG, HRP-linked	Cell Signaling Technology	Cat# 7074
Anti-Mouse IgG, HRP-linked	Cell Signaling Technology	Cat# 7076
Antibodies - RPPA	This paper	https://datashare.ed.ac.uk/handle/10283/9113
Antibodies – Cytokine array	This paper	https://datashare.ed.ac.uk/handle/10283/9113

**Chemicals, peptides, and recombinant proteins**		

Prestwick FDA approved Drug Library	Prestwick Chemical	https://www.prestwickchemical.com/screening-libraries/prestwick-chemical-library/
LOPAC (Library of Pharmacologically Active Compounds)	MERCK/Sigma Aldrich	Cat# LO4100
TargetMol Anti-cancer (2018)	TargetMol	Cat# L2110
Kinase Chemogenomic Set (KCGS)	Structural Genomics Consortium	https://cancertools.org/the-kinase-chemogenomic-set-kcgs/
Comprehensive Anti-Cancer Compound Set (C3L)	Custom Library (Collaboration)	Athanasiadis et al.^45^
Validated/Resupplied Compounds (348 compounds)	This paper	Table S6
Gedatolisib/PF-05212384/PKI-587	Stratech	S2628-SEL
AZD-5305/Saruparib	Stratech	S9875-SEL
OTSSP167	Stratech	B1321-APE
Abemaciclib	Stratech	S5716-SEL
Hoechst 33342	Molecular Probes	H1399
Concanavalin A Alexa Fluor 488	Invitrogen	C11252
SYTO 14	Invitrogen	S7576
Phalloidin Alexa Fluor 594	–	ab176757
Wheat germ agglutinin Alexa Fluor 594	Invitrogen	W11262
MitoTracker DeepRed	Invitrogen	M22426

**Critical commercial assays**		

NucView-488 caspase 3 detection reagent	Biotium	Cat# 30029
Nanostring Human Cancer Pathways Panel	Nanostring	Cat# NS_cancerPath_C2535
RNeasy Plus miniprep extraction kit	Qiagen	Cat# 74134

**Deposited data**		

Raw Image quantification (CellProfiler output, by compound library, includes validation set))	This paper	https://datashare.ed.ac.uk/handle/10283/9113
Processed Data (StratomineR normalized output, redundant feature removal, no dimensionality reduction) w/metadata/plate maps	This paper	https://datashare.ed.ac.uk/handle/10283/9113
HTS QC data (Z-prime by plate, normalized distributions)	This paper	https://datashare.ed.ac.uk/handle/10283/9113
Antibody Lists from RPPA and cytokine arrays	This paper	https://datashare.ed.ac.uk/handle/10283/9113
R script for normalized AUC generation	This paper	https://datashare.ed.ac.uk/handle/10283/9113

**Experimental models: Cell lines**	This paper	https://datashare.ed.ac.uk/handle/10283/9113

Human glioma stem cell: GCGR-E13 (classical subtype, IDH^wt^, EGFR^gain^)	Glioma Cellular Genetics Resource	gcgr@ed.ac.uk, https://github.com/GCGR
Human glioma stem cell: GCGR-E28 (classical subtype, IDH^wt^, EGFR^ecDNA^)	Glioma Cellular Genetics Resource	gcgr@ed.ac.uk, https://github.com/GCGR
Human glioma stem cell: GCGR-E21 (mesenchymal subtype, IDH^wt^, EGFR^gain^)	Glioma Cellular Genetics Resource	gcgr@ed.ac.uk, https://github.com/GCGR
Human glioma stem cell: GCGR-E57 (mesenchymal subtype, IDH^wt^, EGFR^gain^, NF1^mut^)	Glioma Cellular Genetics Resource	gcgr@ed.ac.uk, https://github.com/GCGR
Human glioma stem cell: GCGR-E31 (proneural subtype, IDH^wt^, EGFR^mut^)	Glioma Cellular Genetics Resource	gcgr@ed.ac.uk, https://github.com/GCGR
Human glioma stem cell: GCGR-E34 (proneural subtype, IDH^wt^, EGFR^gain^)	Glioma Cellular Genetics Resource	gcgr@ed.ac.uk, https://github.com/GCGR

**Software and algorithms**		

CellProfiler Image Analysis Software v3.1.5	Broad Institute	Cellprofiler.org: Carpenter et al.^42^; Bray et al.^30^; Bray et al.^38^; McQuinn et al.^43^
CellProfiler Pipeline	This paper	https://datashare.ed.ac.uk/handle/10283/9113
StratomineR high content analysis platform (Cloud)	Core Life Analytics	Stratominer.com
TIBCO Spotfire Analysis Software	Revvity Signals	Spotfire.com
STRING network analysis	STRING Consortium	https://string-db.org/cgi/input?sessionId=blU7e6Xoh7rC
ImageJ 1.54g	Wayne Rasband et al., National Institutes of Health, USA	http://imagej.org
Cytoscape Network Data Integration 3.10.3	Cytoscape	Cytoscape.org
Morpheus (Hierarchical Clustering)	Broad Institute	https://software.broadinstitute.org/morpheus
R Studio (AUC quantification, Figure 3B)	Posit PBC	https://datashare.ed.ac.uk/handle/10283/9113
MetaXpress (Cell cycle analysis)	Molecular Devices	moleculardevices.com

### Experimental model and study participant details

#### Patient-derived glioma stem cells

Glioblastoma cell lines were obtained from the Glioma Cellular Genetics Resource, Edinburgh (gcgr@ed.ac.uk, https://github.com/GCGR). Cell lines were submitted for STR profiling (ECACC, case number 21579, using a Promega Powerplex 16 HS kit) and were routinely monitored for mycoplasma infection (MycoAlert, Lonza). The GCGR patient resource is anonymized, however, sequencing reveals 2 cell lines are male and 4 are female. Cells are cultured on laminin-1 coated substrates, under defined media conditions that have previously been demonstrated to maintain their stem cell-like properties.^108^ Briefly, cells were cultured in DMEM/HAMS-F12 media (Merck, D8437) with B27, N2 supplements (GIBCO 17504-044, 17502-048), EGF/FGF (10 ng/mL, Peprotech 315-09, 100-18b)), glucose (8 mM, Merck, G8644), MEM non-essential amin acids (5 mL, GIBCO 11140-035, 100X), BSA (0.012%, GIBCO 15260-037), β-mercaptoethanol (0.1 mM, GIBCO 31350-010) and laminin (4-10 μg/mL depending on the cell line, Cultrex 3446-005-01). Cells were passaged at ∼80% confluence (4–7 days) and dissociated using Accutase (Merck, A6964). Doubling times are approximately 60 h (except E57 cells, doubling time ∼24 h) with generally low passage ratios (1:3 to 1:6) to maintain no less than 20% confluence during cell expansion. All cell lines were expanded and banked in liquid nitrogen (∼24 vials each) for continuous turnover of low passage lines during screening.

### Method details

#### Compound screening

384-well plates (Greiner Bio-One, μClear, 781091) were precoated in growth media containing 10ug/mL laminin (20μL/well) and incubated for 2 h at 37°C, 5% CO_2_ (minimum). Single cell suspensions in growth media were prepared (final seeding densities indicated in Figure S1B) and added to the pre-coated plates (30μL/well, final laminin 4 μg/mL). The plates were then incubated for ∼20 h and inspected briefly, prior to compound addition using a Biomek automated liquid handler. Compound libraries are described in Table 1. The objectives of each library chosen are as follows: Prestwick FDA: U.S. Food and Drug Administration (FDA) approved drugs, mostly off-patent, suitable for drug repurposing opportunities. TargetMol-Anti-Cancer: Approved anti-cancer agents for non-GBM indications, including more recent targeted therapies suitable for drug repurposing. LOPAC: A well characterized library of marketed drugs, failed development candidates, and reference tools for exploring target biology and drug repurposing opportunities. Comprehensive Anti-Cancer Compound Library (C3L): A library of highly selective tool compounds against known oncology targets to identify new therapeutic target and drug repurposing opportunities for GBM. Compound library plates (as assay ready daughter plates in 100% dimethyl sulfoxide (DMSO), 10mM–1mM stocks, prepared via BioAscent compound management (bioascent.com)) were diluted with media to intermediate plates (1:50, 2% DMSO (v/v)) prior to addition to cells (1:20, final 0.1% DMSO (v/v)). The intermediate drug plates were further diluted to create reduced dose intermediate plates (with 2% DMSO (v/v)) prior to addition to cells (1:20, final 0.1% DMSO (v/v), *n* = 1 per dose, per compound – as is standard in high-throughput screening). Negative controls (DMSO, 0.1% (v/v), *n* = 32–48 per plate) and positive controls (staurosporine, 1 μM final conc., *n* = 16) for cell death were added to the intermediate compound plates (up to 320 compounds per plate). After the addition of compounds, the plates were then incubated at 37°C, 5% CO_2_ for 72 h. Compound resupply details are provided in Table S6.

#### Cell painting

Live mitochondrial staining of cells was carried out by the addition of MitoTracker Deep-Red solution (3 μM, 10X conc. in media at 5μL/well) using the Integra Viafill automated dispenser (Intergra Biosciences). The plates were then incubated for 30 min at 37 °C, 5% CO_2_ followed by plate cooling and fixation by the addition of 15% formaldehyde in phosphate buffered saline (PBS) (20μL/well – final 4% formaldehyde in 75 μL volume) using a multidrop combi reagent dispenser. After incubation for 20 min, the plates were washed (BioTek plate washer, Agilent, www.biotek.uk.com) with PBS and cell painting reagents prepared in 1% BSA/0.01% TX-100 in PBS (Table 2). After washing, PBS was removed and cell painting solution was added using a multidrop combi (20μL/well) followed by incubation at room temperature for 30 min. The staining solution was then removed, the plates washed with PBS, sealed (Starseal foil seals, Starlab, E2796-9792) then imaged immediately.

**Table 2:** Cell painting reagents listed by target organelle, channel, final concentration and supplier details

Stain	Structure	Wavelength, ex/em (nm)	Channel	Final concentration	Catalog no., supplier
Hoechst 33342	Nuclei	387/447	DAPI	5 μg/mL	H1399, Molecular Probes
Concanavalin A Alexa Fluor 488	Endoplasmic reticulum	462/520	FITC	33 μg/mL	C11252, Invitrogen
SYTO 14	Nucleoli	531/593	CY3	3 μM	S7576, Invitrogen
Phalloidin Alexa Fluor 594	F-actin	562/624	TxRED	7 U/mL (1:1,500)	ab176757, Abcam
Wheat germ agglutinin Alexa Fluor 594	Golgi and plasma membrane	562/624	TxRED	3 μg/mL	W11262, Invitrogen
MitoTracker DeepRed	Mitochondria	628/692	CY5	300 nM	M22426; Invitrogen

#### Image acquisition

Images were acquired using ImageXpress Confocal HT.ai high content microscope (Molecular Devices LLC, version 6.5.0) with automated plate handling (GX series, Peak Analysis and Automation). Exposure settings for each channel were optimised and set for screening. Each well was imaged using a 20× objective and *n* = 6 fields of view (30 images per well over 5 channels, ∼33% well coverage, typically 75–150 cells per image) collecting 11,520 images per 384-well plate. Each compound library was screened separately across all six GCGR cells lines. Briefly, cell lines were seeded (500–1500 cells per well) onto laminin pre-coated 384-well plates, followed by compound addition at 24 h using automated liquid handling platforms. DMSO was used as a negative (compound vehicle) control (*n* = 32–48 wells per plate minimum) with staurosporine (1 μM, *n* = 16) typically used as an assay landmark control for cell death. After 72 h compound exposure, cells were subjected to live cell labeling of mitochondria with MitoTracker, followed by fixation, permeabilization, and additional staining with the remaining Cell Painting dyes (see Materials & Methods).

#### Live cell imaging: Proliferation/apoptosis assays

GCGR-E13 (1000 cells/well) and GCGR-E57 cells (500 cells/well) were seeded (45μL/well) onto 384-well plates (Greiner μClear, 781910) pre-coated with 10 μg/mL Laminin ECM. After overnight incubation, a solution of NucView-488 reagent (10X conc., 50 μM) was added (5μL/well). The compounds were then immediately added using a D300e nanodispenser with back-filling of DMSO to 0.1% (v/v) for all wells, triplicate wells per dose (*n* = 3 per compound, based on individual ∼ IC_80_ range). Staurosporine (300 nM, *n* = 6) was used as a positive control for apoptosis induction against DMSO controls (0.1%, *n* = 6). The plate was then loaded into an IncuCyte S5 Live Cell imager with scans set at 3-h intervals for 96 h (phase and ‘green’ channels, 10× objective, 1 site). Image processing was carried using IncuCyte in-built automated image-analysis modules, with phase area defined as % Confluence/image from the first scan (∼t = 0 + 30mins) to quantify cell proliferation over 96 h. Apoptosis was quantified from images showing increasing integrated intensity from the caspase activity biosensor (NucView-488) emission signal (GCU x μm^2^/Image). Statistical analysis at 72 h was carried out using multiple comparison tests for each cell line, via Ordinary one-way ANOVA with correction for multiple comparisons using Dunnett’s statistical hypothesis test (GraphPad Prism, v10.6.0). Data represents best of *n* = 3 biological replicates, presented as mean ± stdev.

#### Western blotting: HDAC and CDK9 inhibitors on GCGR cells

Cells were seeded into laminin (10 μg/mL) coated 10 cm dishes (1 × 10^6^ cells, GCGR-E13 & GCGR-E57 cell lines) and incubated overnight at 37°C, 5% CO_2_. Compound dilutions in media were prepared at 100 nM concentration for all three HDAC (fimepinostat (CUDC-907), Panobinostat, romidepsin) and CDK9 (dinaciclib, NVP-2, AZD4573) inhibitors with 0.1% DMSO (v/v) final concentration. Two DMSO controls (0.1% (v/v)) were used for each cell line and two biological replicates were prepared for each condition/lysate. After addition of each solution, the cells were incubated for a further 24 h at 37°C, 5% CO_2_, followed by washing in cold PBS and lysis with RIPA buffer (Merck, #20–188). Cell lysates were collected, centrifuged (max rpm, 10 min, 4°C) and quantified using Bradford reagent (Thermo Scientific, #1856210) and measuring absorbance (A_595_) benchmarked against Albumin Standard calibration (Thermo Scientific, #23209). Samples were standardised to 1 μg/μL in RIPA buffer and denatured by heating to 95°C with Laemmli sample loading buffer (Bio-Rad, #1610747) and dithiothreitol (DTT, 50 mM) for 5 min. Samples (20μg/well) were loaded onto Bio-Rad pre-cast PAGE gels (Bio-Rad, mini-Protean TGC gels 4–20%, #4568095) using SeeBlue Plus2 pre-stained standard M.W. ladder (Invitrogen, #LC5925) followed by transfer to PVDF membrane (Bio-Rad, *Trans*-blot Turbo pack, #1704156) and blocking with 5% BSA/TBS-Tween (0.1% (v/v)). All antibodies used are listed in STAR Methods key resources table (1:1000 dilutions, note: multiple gels were run for the various antibody probes used) and used HorseRadish Peroxidase (HRP) conjugated secondary antibodies. Blotting membranes were stripped for re-probing using Re-Blot Plus 10X solution (Merck, #2504). All blots were imaged from chemiluminescent detection (Roche, #11500694001) using a Bio-Rad Gel-Doc XR + system and Image Lab software (v6.1.0 build 7).

#### NanoString sequencing/differential analysis

mRNA was extracted from E13 cells (∼3 × 10^6^ cells each condition, *n* = 3) after 24 h treatment with AZD473, Dinaciclib, NVP-2 (all 20 nM) & DMSO (0.1% v/v) in triplicate 10 cm dishes, using RNeasy Plus miniprep extraction kit (Qiagen, #74134). Purified mRNA was quantified and diluted to 20 ng/μL and submitted for NanoString™ nCounter® analysis against the Human Cancer Pathways Panel (NS_CancerPath_C2535). Samples were hybridised and immediately processed using the nCounter Prep Station and Digital Analyzer (High Sensitivity protocol). QC checks utilised GeNorm to determine most stable reference genes and no QC Flags were raised overall. Differential analysis was carried out on normalised data (normalised to ∼20 housekeeping genes) using nSolver Analysis Software (version 4.0.70) following standard analysis pipelines (nCounter Advanced Analysis plugin, version 2.0.134, with corrected *p*-value to control the false discovery rate (FDR, Benjamini-Yekutieli). Exported data was replotted using TIBCO Spotfire software and also further analyzed by hierarchical clustering (https://software.broadinstitute.org/morpheus) and STRING network analysis (string-db.org).^109^ Venn diagrams were generated from Molbiotools (molbiotools.com/listcompare.php).

#### Forward-phase protein array: Basal cytokine analysis

Conditioned medium was collected after 72 h incubation (*n* = 4 per cell line). Microarrays were generated using an in-house Aushon BioSystems 2470 array printing platform. The arrays were blocked for 1 h with Superblock T20 Blocking Buffer (Grace Bio Labs) at room temperature. Conditioned media from glioma stem cell samples were centrifuged at 1000× *g* for 5 min at 4 °C. Supernatants were collected and added to microarrays followed by incubation overnight at 4 °C. The arrays were washed three times for 5 min in PBS-T (PBS-Tween 0.1% v/v) and blocked for 10 min with Super G Blocking Buffer at room temperature on a rocking platform, then washed again washed three times for 5 min in PBS-T. Detection antibody mixtures (1:500 antibody dilutions in 5% bovine serum albumin/phosphate buffered saline tween 20 0.1% (BSA/PBS-T), 10% Super G Blocking Buffer) were made in microplates. Microarrays were clamped and 50 μL of each antibody was added to corresponding microarray wells. Microarrays were incubated for 1 h on a rocking platform. Clamps were removed and microarrays were washed three times for 5 min in PBS-T. Microarrays were then blocked for 10min with Super G Blocking Buffer at room temperature and again washed three times for 5 min in PBS-T. Three millilitres (3 mL) of IRDye 800CW Streptavidin (LI-COR Biosciences) was diluted 1:5000 in PBS-T supplemented with 5% BSA, 10% SuperG Blocking Buffer. Microarrays were covered and incubated with IRDye on a rocker at room temperature for 30 min, then washed for 5 min, three times in PBS-T followed by three 5-min PBS washes and finally washed with distilled water. Microarrays were dried then scanned at 795 nm on the InnoScan 710 high-resolution microarray scanner (Innopsys Life Sciences). The data was normalised for protein concentration and background fluorescence via Microsoft Excel templates.

#### Reverse-phase protein array (RPPA): Basal analysis

GSC lines were cultured both as mono-layers on laminin-coated cell culture plates and as spheroid cultures on poly-hema coated plates (*n* = 1 per cell line, per condition). Cells were set up as mono-layer and spheroid cultures in parallel by seeding equivalent cell numbers from a parental flask with cells in log-phase growth. Cultures were grown until the mono-layer cultures were approximately 70% confluent and then both adherent and suspension cultures were harvested for lysis and preparation for RPPA. For Laminin coating, plates were incubated with Laminin at 10ug/ml for 1 h and then washed with PBS prior to addition of cell lines. Culture media did not contain Laminin and the same media was used for adherent and suspension cultures. For Poly-hema coating plastic 2.4g of poly-hema was dissolved in 200 mL of 95% Ethanol and heated to dissolve. The solution was centrifuged at 2500RPM to remove undissolved particles. 4 mL was added per 10 cm plate (ensuring the plate was completely coated). Plates were left open in a TC hood to air-dry and were rinsed x3 with PBS prior to addition of cells.

Mono-layer cultures were washed twice with PBS after removal of media and snap frozen on dry-ice and stored at −80°C. Spheroid cultures were pelleted by centrifugation and washed twice with PBS and then snap frozen and stored until lysis. Cultures were lysed in RIPA buffer supplemented with ROCHE PhosStop and cOmplete Mini inhibitor tablets (Merck, 04906845001 & 11836153001). Lysates were needle syringed five times to ensure complete lysis of cells. Protein quantification was carried out using Coomassie-plus assay (ThermoFisher #23228) and protein concentration was adjusted by to 1 mg/mL. Samples were prepared for printing by addition of 4x Sample buffer and heat denaturation at 95°C for 5 min.

All samples were diluted to 0.75 mg/ml (D1), 0.375 mg/ml (D2), 0.1875 mg/ml (D3) and 0.09375 mg/ml (D4) using PBS containing 10% Glycerol. Array spotting was carried out with the Quanterix 2470 Arrayer platform using 185 μM pins. Each sample was spotted at four dilutions (D1-D4) onto single pad ONCYTE SuperNOVA nitrocellulose slides at a 500 μM spot-to-spot distance. 268 signaling pathway markers (and appropriate controls) were profiled in a standard RPPA assay as described briefly below. After spotting, the slides were incubated with antigen retrieval solution (1x Reblot strong) for 10 min before being placed in a microfluidic structure to individually address the arrays with primary or secondary antibody solutions. Following blocking buffer (Superblock T20) for 10 min the detection of marker antibodies was performed in a two-step sequential assay (1) incubation of an array with primary analyte-specific antibody in blocking buffer for 60 min at room temperature and (2) removal of excess antibody by washing arrays with PBS-T, followed by a further incubation with blocking buffer and PBS-T washes, and incubation secondary antibody (Dylight-800-labeled anti-species antibodies diluted 1:2500 in Superblock T20) for 30 min. After further washing and slide drying, the arrays were imaged in the Innopsys Innoscan 710 scanner. Blank signals were determined by omitting the primary antibody from step 1 and instead incubating the array with Superblock T20 alone, followed by step 2. Sample loading on arrays (for normalisation) was determined by staining one slide with fast green protein dye and scanning at 795 nm. Microarray images are analyzed using Mapix software (Innopsys). The feature (spot) diameter of the grid was set to 270 μm. The average signal intensity is determined for each individual feature and the median background from the adjacent area is subtracted from each feature signal leading to a net signal per feature. Data analysis is performed in a standard way: Fluorescence intensity for each feature on the array is measured. A test is performed for linear fit through the 4 point dilution series for all samples on all arrays using a flag system where R2 > 0.9 (green flag) is deemed good, >0.8 (amber flag) is deemed acceptable and <0.8 (red flag) is poor and may be excluded from data analysis. The median values from the 4 point dilution series are calculated and used as a measure of fluorescence intensity. Data is quantified as RFI (relative fluorescence intensity) values relating to relative abundance of total and phosphorylated proteins across the sample set.

### Quantification and statistical analysis

#### Image analysis, quantification and data processing/plotting

CellProfiler v3.1.5 image analysis software (cellprofiler.org^30^^,^^38^^,^^42^^,^^43^) was used to create a custom analysis pipeline across 5 channels capturing approx. 1006 features per cell (processed features & factor loadings/explained variance is provided in Table S7, the custom CellProfiler pipeline is also provided: https://datashare.ed.ac.uk/handle/10283/9113). Images were analyzed via parallel processing using a Unix/Linux high performance computing cluster (University of Edinburgh) and concatenated outputs included median aggregated, well level data (*n* = 6). Each completed dataset (library across 6x GCGR lines) was then batch processed using StratoMineR high content analysis platform (stratominer.com). Briefly, stepwise processing in StratoMineR included metadata integration, removal of redundant features (correlation cut-off 0.99, typically 800–850 features remaining), QC and control annotation/flags, normalisation (to DMSO and sample wells, on a plate-by-plate basis), transformation (Skewness significance 0.0001), feature scaling (robust *Z* score, by plate) and dimensionality reduction (principal component analysis [generalised weighted least squares], Oblique rotation, Ten Berge factor scores method, auto correlation cut-off with ≥20 factors chosen [model based on samples] to represent >60% of data variance [Scree plots] and computation of phenotypic distance/*p*-values from principal component vectors.^46^

We computed the phenotypic distance *p*-values with the embedded R functions from StratomineR from *n* = 6 replicate images per well. The algorithm first computes the Euclidean distance, using the values from each replicate relative to the control wells of each plate. Separate treatment of the plates is necessary to account for plate-to-plate variation and to avoid mixing replicate distributions. To convert distances into statistical significance scores, StratoMineR uses an analytical null model. Specifically, each replicate’s distribution of Euclidean distances is modeled using a Poisson probability distribution, where λ is set to the mean of the observed distance distribution for that replicate. *p*-values are then derived directly from the Poisson cumulative distribution function. No resampling, permutation, or bootstrapping procedures are used in this step. The null model therefore represents the expected frequency of distances under the empirical distribution of the replicate, assuming Poisson behavior. After replicate-wise *p*-values were calculated, we took the median of replicate distances for downstream hit selection. We then used StratoMineR’s Hit Selection module, which includes built-in support for multiple-testing correction. We employed FDR, the default procedure to control the expected proportion of false positives among all statistically significant hits. Under this procedure, the phenotypic-distance *p*-values produced by the Poisson model are adjusted so that, on average, no more than the chosen fraction of selected hits are expected to be false discoveries.

Image quality control (QC) was performed using features generated by the CellProfiler ImageQC module (version 3.1.5). All available ImageQC features were included in the assessment. QC feature values for all images across the six glioblastoma stem cell lines were exported and analyzed collectively without normalization. Principal component analysis (PCA) was conducted in Revvity Signals VitroVivo (version 3.1.0) using the High Content Profiler App Powered by TIBCO Spotfire (version 12.0.3.775). PCA was used to visualize the multidimensional feature space and facilitate the identification of clusters of images with poor quality. Representative images from these clusters were manually reviewed to confirm image quality issues (e.g., defocus, uneven illumination, or saturation). Clusters confirmed as low quality were excluded from downstream analysis. Across the five compound library screens, between 0.7% and 3.0% of images were flagged for removal: TargetMol (2.4%), LOPAC (1.4%), FDA (3.0%), C3L (0.7%), and KCGS (2.5%). This process was performed independently for each screen following a consistent workflow.

Processed data was then exported and analyzed further using TIBCO Spotfire (version 12.0.3.775), including plot generation. Hierarchical clustering of normalised datasets was carried out using Morpheus (https://software.broadinstitute.org/morpheus) by complete linkage, Euclidean distance). Network and enrichment analysis of top 45 [supplier] annotated compound targets was carried out using STRING^109^ & Cytoscape (cytoscape.org).^110^ Cell cycle analysis was carried out from DNA content/nuclei images (Hoechst stain) using MetaXpress software (Molecular Devices LLC, version 6.5.0). Dose response data and curve fitting (non-linear regression) was carried out using GraphPad Prism (v7.0.5). Synergy plots were generated using SynergyFinder+ web application^111^ (https://synergyfinder.org/).

#### Clustering analysis and distribution plots

To quantify the phenotypic diversity of the screened compounds with *Z* score [cell survival] < −3 and -Log_10_[*p*-value] > 2 we performed *k*-means clustering of the unique 245 compounds that passed this threshold using the principal components derived from the morphological features. The degree of clustering was quantified by the *k*-means score defined as the within-cluster sums of point-to-centroid distances, summed across all clusters.

$$sum-of-squares\;criterion=\sum_{j=1}^{K}\sum_{n\epsilon S_{j}}\left({\left\|x_{i}-\mu_{j}\right\|}^{2}\right)$$

Where *n* = total number of data points, *K* = number of predefined disjoint clusters *S*_*j*_, x_n_ = vector representing the nth data point and μ_j_ = geometric centroid of the data points in *S*_*j*_.

A lack of inflection point at any given *k* is indicative of the compounds’ loose attachment to their assigned clusters i.e., a lack of defined morphological profiles in the selected drug treatments. Clusters were varied from 2 to 20, with a maximum of 10 iterations allowed, and a random initial set allocation of 25 distinct dataset rows. The quality of the *k*-means clusters was determined with the silhouette coefficient *S* averaged across the unique compounds per library.^47^

$$s\left(i\right)=\frac{b\left(i\right)-a\left(i\right)}{\max\{a\left(i\right),b\left(i\right)\}}$$

Where for each *i* point in the dataset, *b(i)* = distance between *i* and its next nearest cluster centroid and *a(i)* = mean distance between *i* and all other cluster members.

The silhouette coefficient varies between −1 and 1, with *S* = 1 indicating that compounds are in well separated clusters, *S* = 0 indicating overlapping clusters, and *S* = −1 indicating incorrect assignment of clusters. The distribution of the variable -Log_10_[*p*-value]-Phenotypic Distance was calculated using violin plots per cell per library. These measures were calculated using R version 2023.9.0.463 with libraries base stats (function kmeans), cluster_2.1.4 (function silhouette) and ggplot2_3.4.3 (function geom_violin).

#### NanoString nCounter analysis

NanoString nSolver Analysis Software (version 4.0.70) used nCounter Advanced Analysis plugin (version 2.0.134) for differential expression analysis. *p*-values from normalised data (normalised to ∼20 housekeeping genes) and triplicate sample data was then corrected to control the false discovery rate (FDR, Benjamini-Yekutieli).

#### GraphPad prism statistics

GraphPad Prism (v7.0.5 to v10.6.0) (Figures 5C–5F and 6B–6E) utilised Ordinary One-Way ANOVA with multiple comparison tests for each cell line (and correction for multiple comparisons using Dunnett’s statistical hypothesis test). Dose response data (Figures 5B and 6A) was generated from DMSO normalised data [with Log transformed concentrations] from non-linear regression analysis (LOG-Inhibitor vs. Normalised data – Variable slope [model Y = 100/(1 + 10ˆ((LogIC50-X)∗HillSlope))]). Inhibitory concentration at 50% survival/death (IC_50_) values represent ‘best fit’ values with 95% Confidence Interval ‘[dose response] profile likelihood’. Dose response data (post-validation) represents a minimum of *n* = 3 biological replicates.

#### Similarity matrix [combined IC_50_, basal RPPA and cytokine data]

Similarity matrix (Figure S7; Table S4) was generated using Morpheus matrix visualisation and analysis software (https://software.broadinstitute.org/morpheus) using Spearman Rank Correlation and Hierarchical Clustering (One minus Spearman Rank Correlation, Complete Linkage method).

#### STRING-cytoscape network enrichment analysis

Gene Lists used to generate String/Cytoscape figures (Figures 3 and 7; Figures S2, S6 and S10–S12) and enrichment analyses (Tables S3 and S5) used parameters: Filtering: −1.6 > Log2(Fold-Change) > 1.6; Corrected *p*-value (Benjamini-Yekutieli FDR <0.05); STRING settings: Normal Geneset analysis, no text mining, query proteins only, med-high confidence interactions (0.400–0.700).

## Supplemental information

Document S1. Figures S1–S17

Table S1. Details of 164 validated compounds with target annotation, prioritization and BBB permeability predictions (in Figures S and electronic version also provided)

Table S2. (i) dose response validation dataset (ii) alphabetical list of most significant hits by p value (iii) quality control data included Z-prime factor and normalized distributions (iv) SMILES data and BBB permeability prediction across purchased validation set, relating to Figure 2, Figure 3

Table S3. Enrichment analysis from STRING-Cytoscape network of annotated compound target(s) of Top45 prioritized compounds, relating to Figure 3G and Figure S7

Table S4. Complete dataset of similarity matrix from combined IC50, basal RPPA and cytokine data, including full similarity matrix, correlation matrix by compound name and target annotation, re-scaled datasets used in the similarity matrix plus original (normalized) data, relating to Figure S7

Table S5. CDK9 inhibitors; complete NanoString analysis (differential expression, unfiltered with FDR values) plus enrichment tables from STRING analysis from top ranked genes (from Figure 7B, C) and Venn diagram data from Figure 7C, relating to Figure 7 and Figures S10–S12

Table S6. Details of the compounds purchased for all validation steps and for the HDAC inhibitor bespoke set, with supplier/catalog numbers, relating to Figure 3

Table S7. Feature loadings across all principal components, relating to Figure 2 A, B, and C
