# Supplementary material for: A comprehensive pharmacological survey across heterogeneous patient-derived glioblastoma stem cell models
Source: iScience. 2026 Apr 27;29(6):115839. doi: 10.1016/j.isci.2026.115839 (PMC13207355; doi:10.1016/j.isci.2026.115839)
Supplement: Document S1. Figures S1–S17 [file mmc1.pdf]

iScience, Volume *29*

Supplemental information

A comprehensive pharmacological survey across heterogeneous patient-derived glioblastoma stem cell models

Richard J.R. Elliott, Peter W.K. Nagle, Muhammad Furqan, John C. Dawson, Vanessa Smer-Barreto, Diego A. Oyarzún, Aoife McCarthy, Alison F. Munro, Camilla Drake, Gillian M. Morrison, Steven M. Pollard, Michael Marand, Daniel Ebner, Valerie G. Brunton, Margaret C. Frame, and Neil O. Carragher

Supplementary Figure 1. Optimisation of seeding densities and incubation A. Cell Line Molecular data (Glioma Cellular Genetics Resource (https://github.com/GCGR). B. Representative phase contrast images of GCGR cells at 72 hrs. GCGR-E13 (classical subtype, 1000 cells/well), GCGR-E28 (classical, 1500 cells/well), GCGR-E21 (mesenchymal, 1000 cells/well), GCGR-E57 (mesenchymal, 500 cells/well), GCGR-E31 (proneural, 1000 cells/well), GCGR-E34 (proneural, 1000 cells/well). Scale bar 400μM. C. Quantification of live cell imaging and growth curves for 384w optimisation. Doubling times ~60 hrs (except E57 cells ~24 hrs) (n=3 replicates and n=3 biological replicates). D Optimisation of seeding density and quality control based on coefficient of variation (%CV) <20% (based on 'Average Number of Nuclei' per site/n=6 fields of view, n=4 biological replicates). Note: The term injury and developmental refer to recent scRNA-Seq studies indicating that GSCs map along a transcriptional gradient spanning two cellular states reminiscent of normal neural development "developmental" and inflammatory wound response "injury".

A

GCGR Cell line ID	E13	E21	E28	E31	E34	E57
Tumour classification	GBM IDH wt	GBM IDH wt	GBM IDH wt	GBM IDH wt	GBM IDH wt	GBM IDH wt
Cell line transcriptional subtype[1]	Classical	Mesenchymal	Classical	Proneural	Proneural	Mesenchymal
Cell line transcriptional subtype[2]	Injury	Injury	Developmental	Developmental	Developmental	Injury
EGFR copy number status	Gain	Gain	Gain ecDNA	–	Gain	Gain
EGFR mutation status	–	–	–	Missense mutation	–	–
NF1 copy number status	–	–	–	–	–	–
NF1 somatic mutations	–	–	–	–	–	Multi-hit

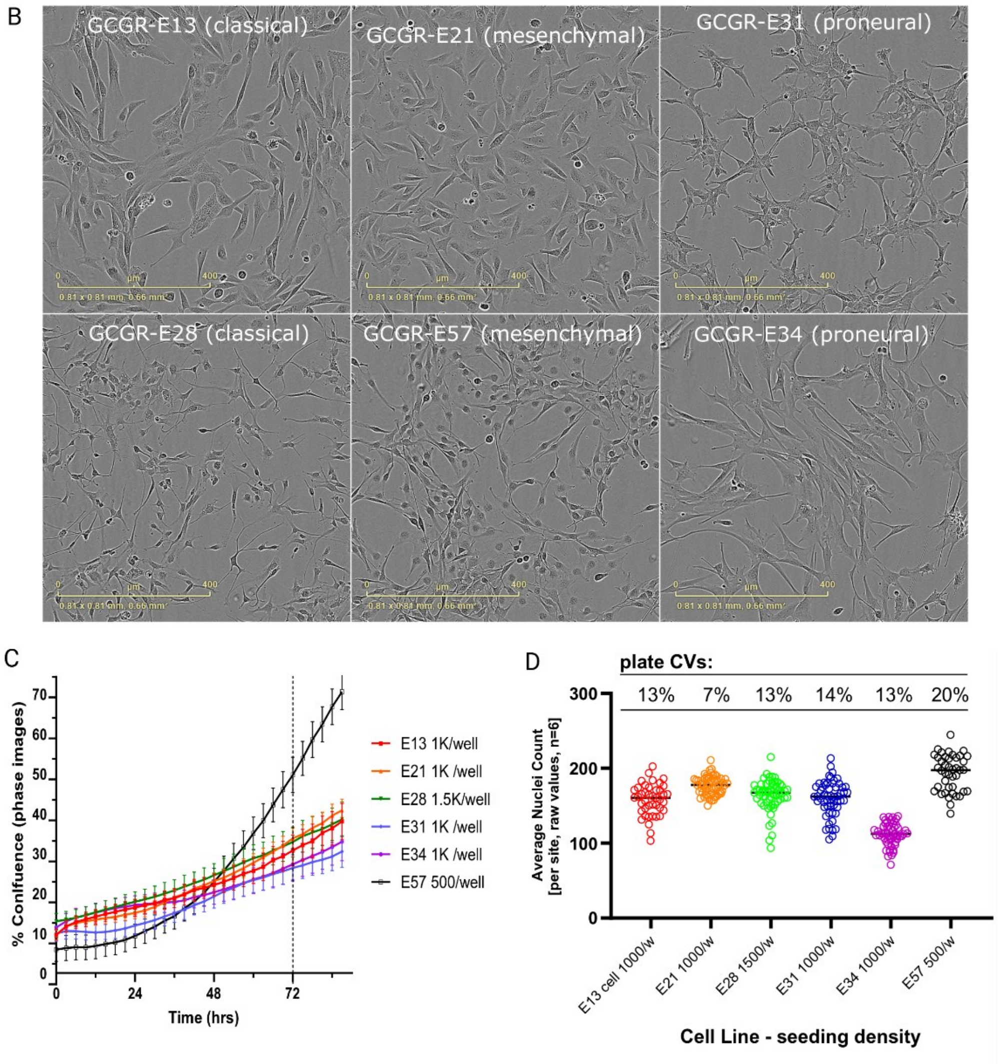

Supplementary Figure 2. Basal characterisation of GSC cell models by reverse phase protein and cytokine array A. Top ranked Z scores of normalised protein levels (268 antibodies inc. controls, n=1 per cell line & condition), with comparison to 2D and 3D conditions. Hierarchical clustering by one minus Pearson correlation and complete linkage. B. Network analysis of basal expression across all cell lines (2D+3D). C. Top ranked Z scores of basal cytokine expression in GSC models (n=4). D Network analysis of high basal expression of cytokines in GCGR cells.

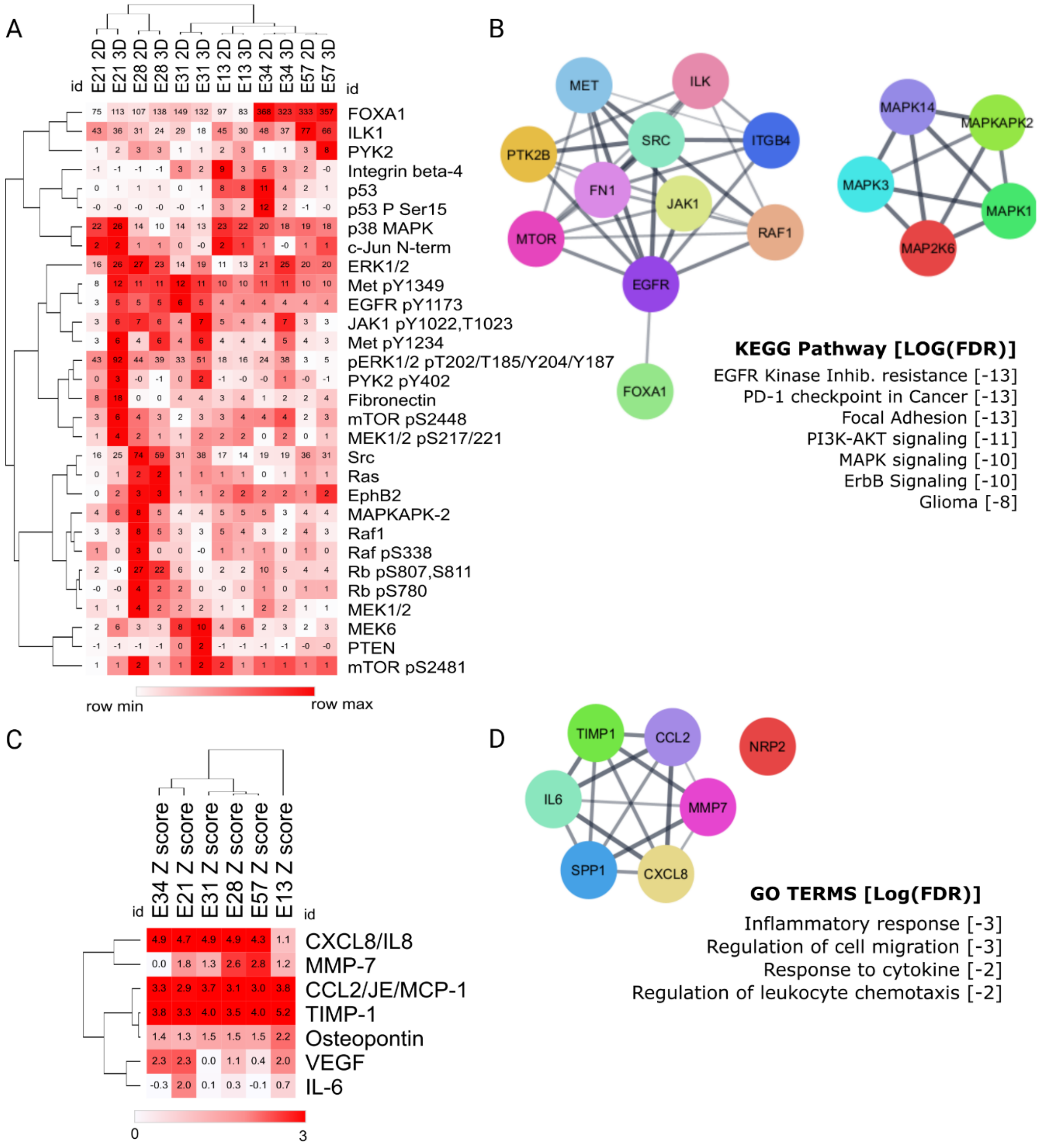

Supplementary Figure 3. Dose response optimisation of cell death on GCGR cells (positive controls, cell death) with IC50 values (nM) and Z prime calculations for 1µM doses. (n=3 replicates, minimum n=3 biological replicates, data points are represented as mean ± stdev)

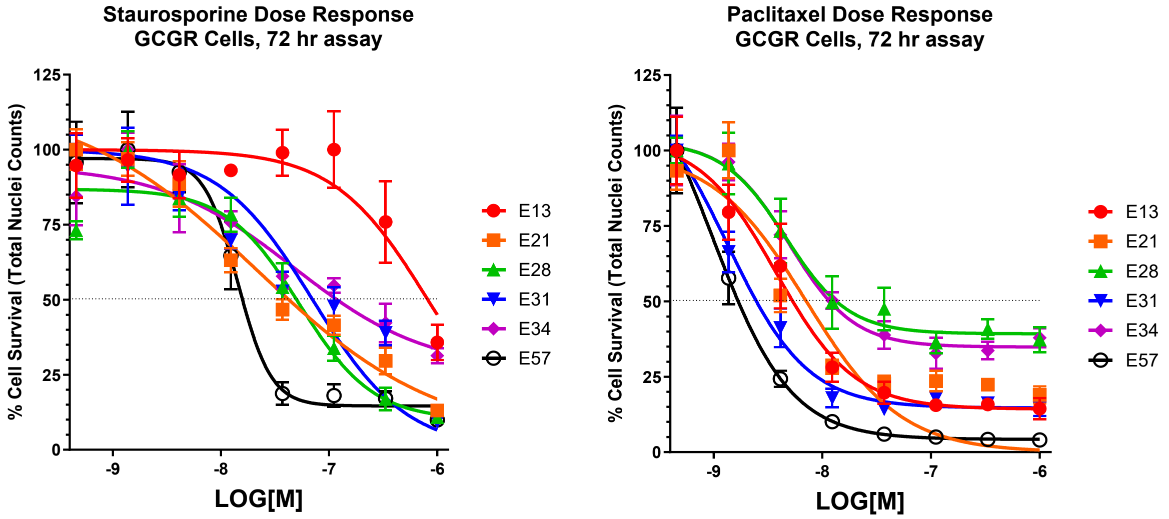

	E13	E21	E28	E31	E34	E57
Stauro IC50 nM	817.7	45	47.98	70.39	129.6	19.74
PAC IC50 nM	6.56	7.124	33.01	3.333	22.74	2.029

Z' factor: 0.1-0.5 [Staurosporine, 1uM]
0.4-0.6 [Paclitaxel, 1uM]

Supplementary Figure 4. Pilot screening data: TargetMol anti-cancer set L2110, 330 compounds (KCGS data excluded): A. Principal component analysis (n=6 fields of view) showing strong phenotypes in red (arbitrary thresholds) and B. Phenotypic distance vs Cell survival (normalised z score). Strongest hits in red, strong phenotypes in purple.

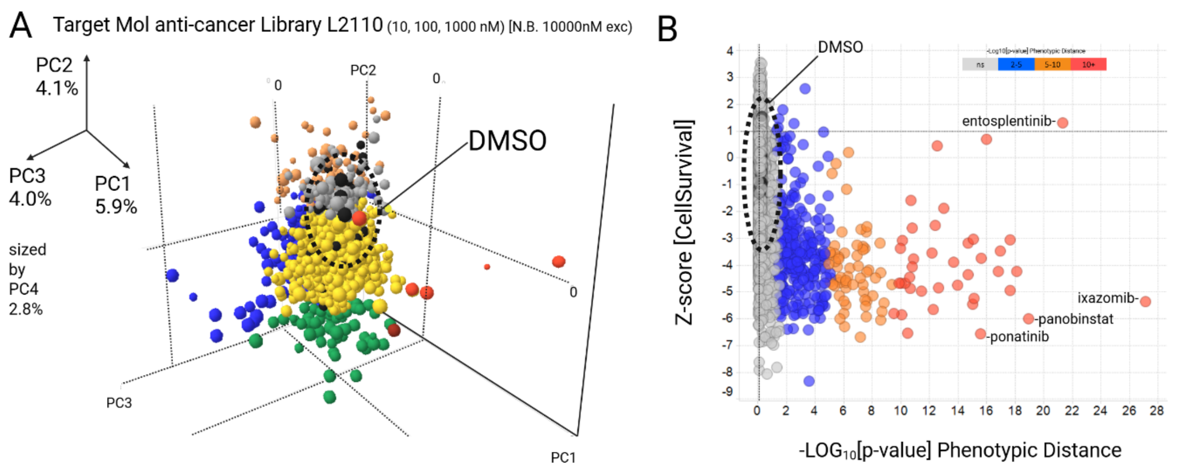

Supplementary Figure 5. Multiparametric phenotypic data analysis of compounds that affect cell viability and phenotypic distance far from DMSO controls[54]. (A, B) Cluster structure of the screened compounds with Z-Score [cell survival] < -3 and -LOG$_{10}$[p-value] > 2 using the principal components derived from morphological features (Figure 2, n=6 fields of view). Plot (A) shows the *k*-means clustering score and plot (B) the silhouette coefficient[55] averaged across compounds for an increasing number of clusters (*k*). Error bars denote one standard deviation over 125 repeats with random initial seeds. The lack of a clear "elbow" in the *k*-means score and low silhouette coefficients suggest poor clustering. (C) Distribution of -LOG$_{10}$[p-value] Phenotypic Distance per chemical library and GCGR cell line screened, for the compounds with the same thresholds as in panels (A) and (B). The colour gradient quantifies -LOG$_{10}$ [p-value] > 2. The heterogeneity of the cell lines is reflected in the diversity of the distributions observed, particularly in the mesenchymal cell lines (GCGR E21 and GCGR-E57).

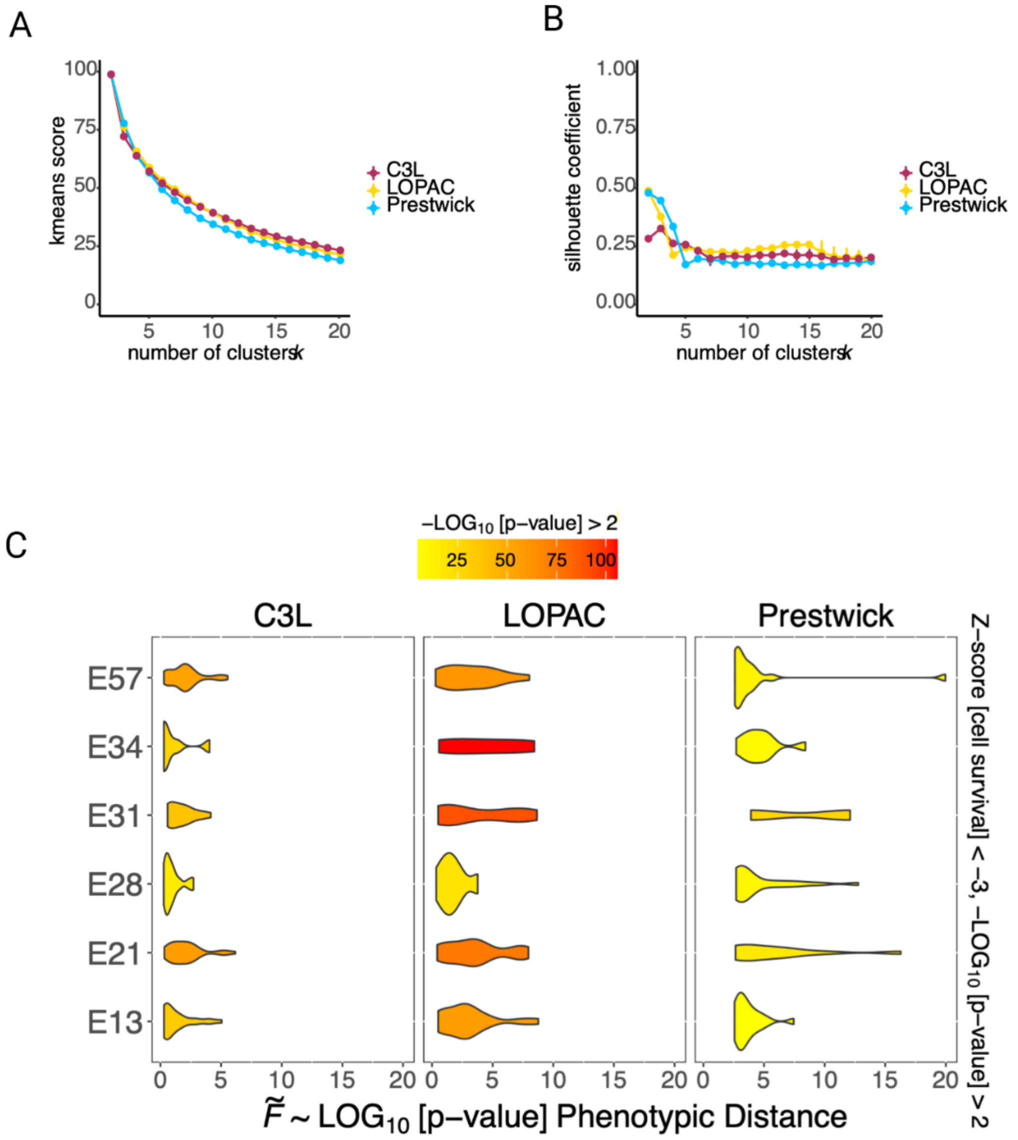

Supplementary Figure 6. STRING-Cytoscape network & enrichment analysis, of the annotated targets of the validated hits, demonstrates broad target-pathway coverage, including PI3K-AKT, MAPK, EGFR & adhesion signalling. B-F. Specific signalling pathways linked to annotated compound targets (highlighted in yellow) with False Discovery Rate [Log[FDR]] ranked GO terms.

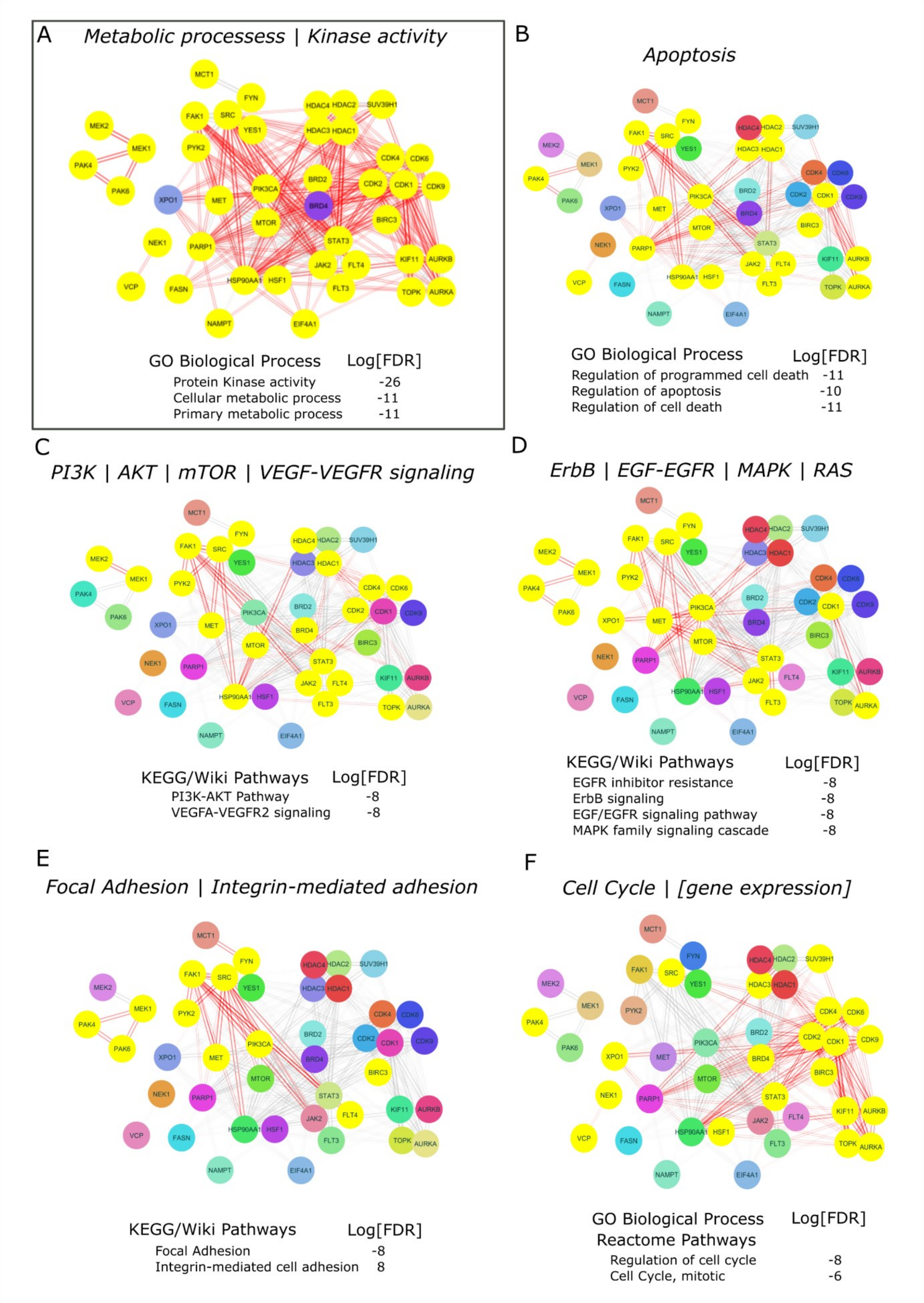

Supplementary Figure 7. Spearman Rank Correlation of combined protein expression data (Reverse Phase Protein array, Cytokine Array, 79 probes) with IC_{50} data (45 validated compounds) across 6 glioma stem cell lines (mixed subtypes, CLA/PRO/MES). All data was re-scaled (0-1) and analysed by Spearman Rank Correlation Similarity matrix and Hierarchical Clustering (One minus Spearman Rank Correlation, Complete Linkage) using Morpheus (https://software.broadinstitute.org/morpheus). A. Full matrix, Spearman Rank Correlation with Hierarchical Clustering, scaled -1 to 1. Positive correlation (RED) suggests protein expression drives compound resistance (higher IC50s) and negative correlation (BLUE) suggests protein expression is driving sensitivity to the compound (lower IC50s). B. Edited Spearman Rank Correlation/Clustering showing Compound (x-axis) vs Protein names (y-axis). C. Selected data showing expression of fibronectin (FN1, top row indicated with arrow) which negatively correlates with FAK1/2 inhibitor (VS4718), SRC inhibition (eCF506, ponatinib, dasatinib), MEK inhibition (trametinib, GDC-0623) [BLUE squares] and strongly, positively correlates with resistance to AZD3695 (top right arrow), an inhibitor of MCT1/SLC16A1 [RED square]. D. Comparison of actual fibronectin signal (RPPA data) with SRC and FAK inhibitor potency (IC50 data) across 6 GCGR cell lines, from which the correlation matrix is derived. Heatmap by ROW effect across cell lines (min-max).

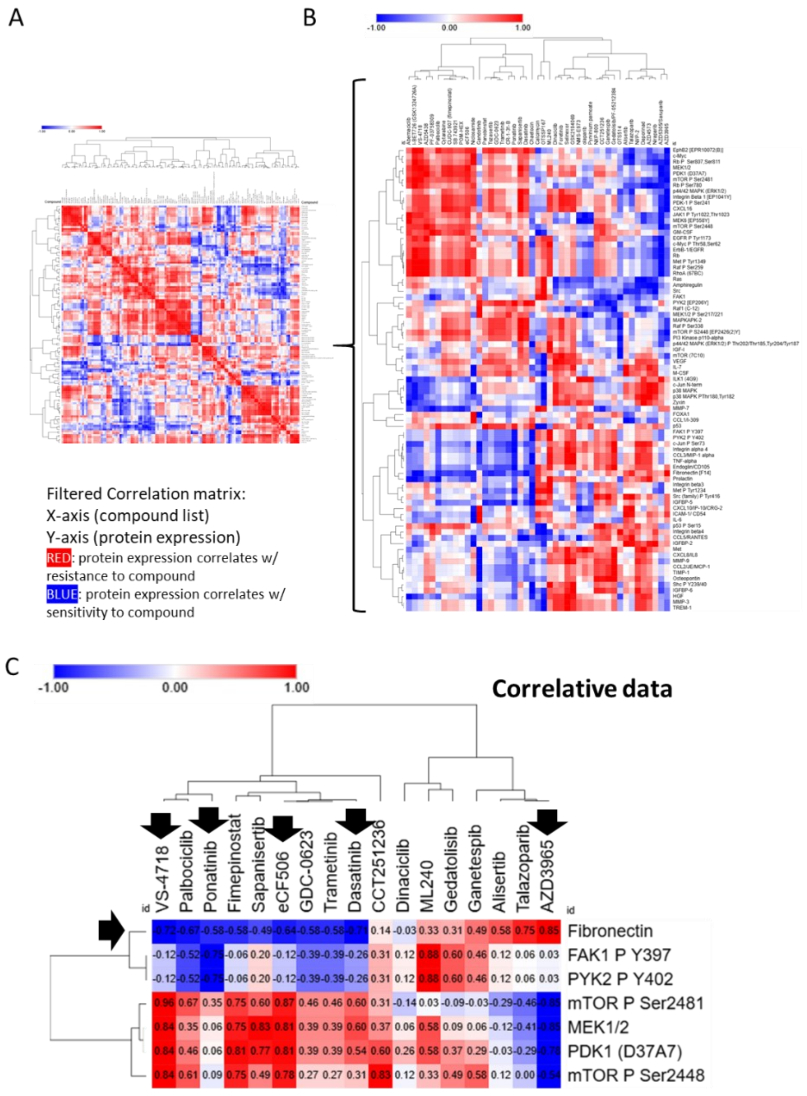

D **Functional data**: Fibronectin expression vs IC_{50} values (SRC, FAK inhibition)

	med FN1	high FN1	low FN1	high FN1	med FN1	med FN1	
ID	E13	E21	E28	E31	E34	E57	
Fibronectin [FN1]	0.183	0.529	0.091	0.249	0.132	0.141	norm FU (RPPA signal)
eCF506 (SRC)	2376	316	7794	208	13860	120	IC50 nM
Dasatinib (SRC, ABL, Kit)	166	79	1474	17	731	32	IC50 nM
Ponatinib (SRC, ABL, PDGF)	2250	501	1006	205	1010	852	IC50 nM
VS-4718 (FAK)	2926	1152	10000	2287	5190	846	IC50 nM

row min — row max

Supplementary Figure 8. Cell Cycle effects of HDAC and CDK9 inhibitors by dose response (%G0/G1 and (%G2/M) plus normalised nuclei counts). Calculated from nuclei stain/images & DNA content analysis (n=3 technical replicates over n=3 biological repeats, data points are represented as mean ± stdev).A to F. Dose response (300nM to 0.6nM) across 6 GCGR cell lines. Percentage cell cycle (left y-axis) with %G0/G1 (red) and %G2/M (blue) with normalised nuclei counts as % cell survival (right y-axis, black). *[cell cycle data excluded where high doses result in complete cell death]*

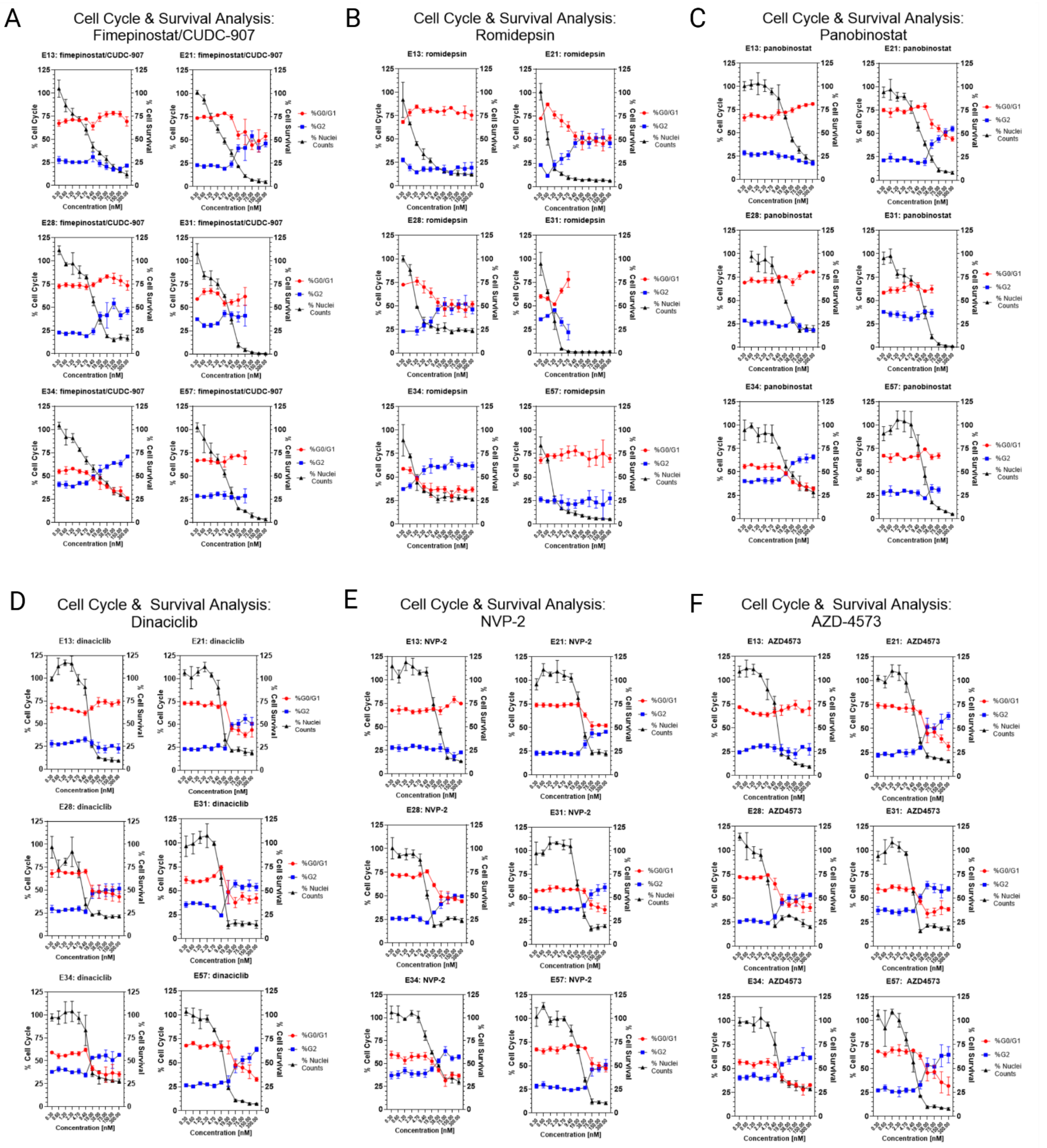

Supplementary Figure 9. Western blot analysis of anti-HDAC activity of fimepinostat (HDACi, PI3Ki, 100nM), Panobinostat (pan-HDACi, 100nM) and romidepsin (pan-HDACi, 100nM) versus DMSO controls (n=4) across two GCGR cell lines, E13 and E57. Cell lysates were prepared over two biological replicates at a 24 hour time point. A. Compound dependent increase in acetyl-histone H3 (K9) relative to DMSO controls (n=2 per cell line). *split panels due to varied exposure times. B&C Total histone H3 and tubulin loading control. D. In a separate blot, phospho-AKT (Serine-473) is slightly reduced in dual PI3K/HDAC inhibitor fimepinostat but not in other specific HDAC inhibitors. E&F. Total AKT and tubulin loading control. *split panels due to varied exposure times

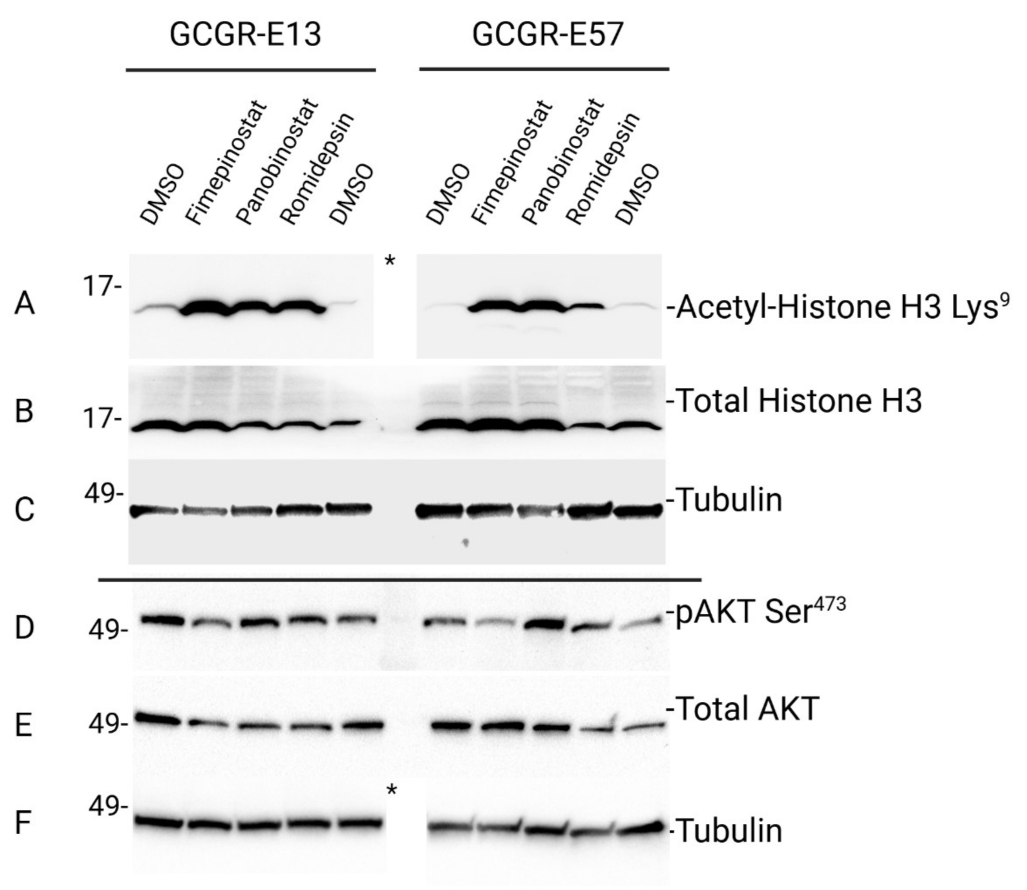

Supplementary Figure 10. Nanostring™ nCounter® transcriptomics and differential analysis of CDK9 inhibitors versus DMSO (0.1% (w/w)) on GCGR-E13 cells (n=3 per sample/condition). A. Network enrichment analysis (string-db.org) of AZD4573, filtered by minimum 3-fold change and FDR <0.05 (277 genes). B. Venn diagram comparison of differentially expressed genes across AZD4573, dinaciclib & NVP-2. With differentially expressed genes unique to AZD4573 indicated. C. Summary of Biological processes (Gene Ontology) enriched in AZD4573 treated, GCGR-E13 cells (string-db.org). Coloured by FDR and sized by gene count.

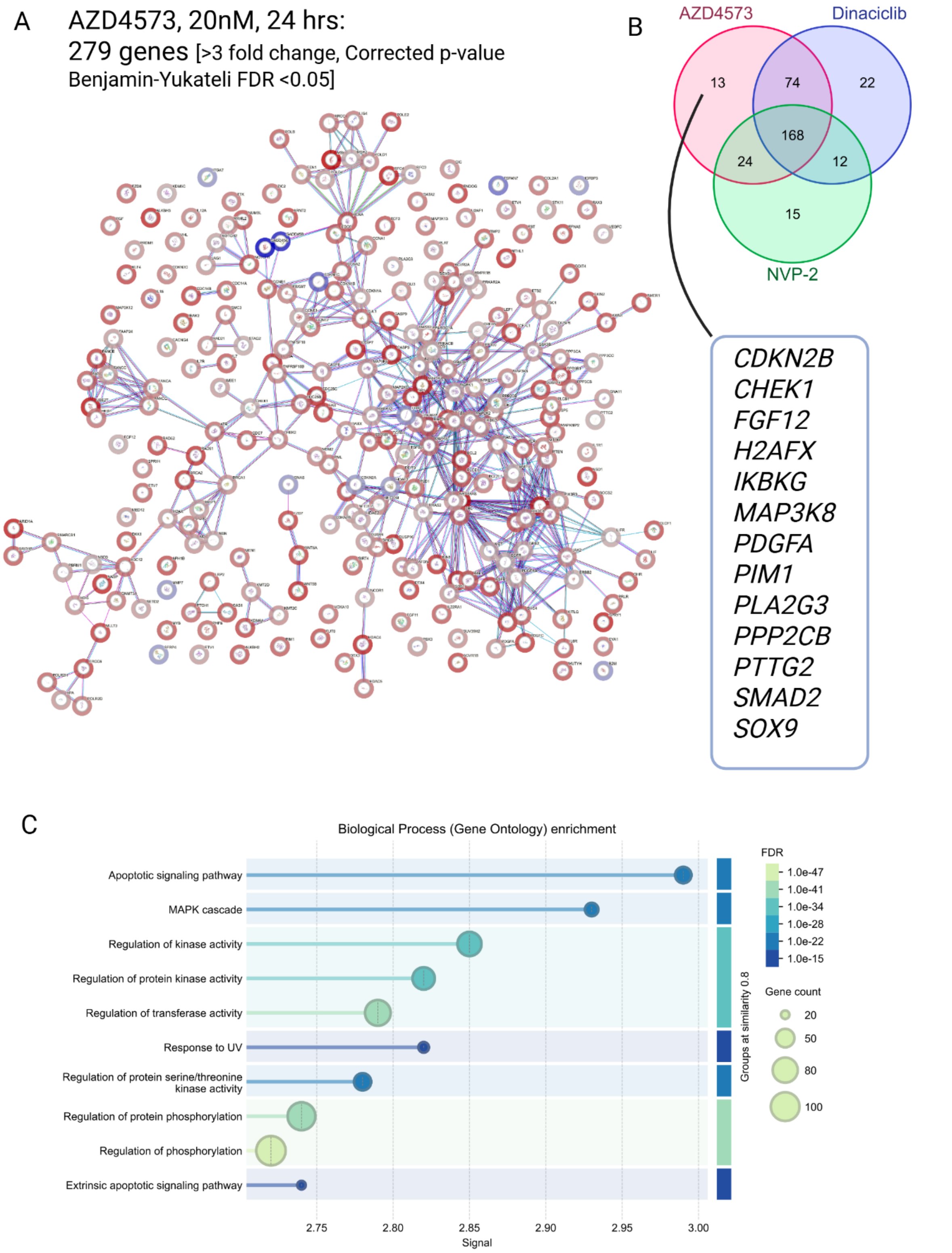

Supplementary Figure 11. Nanostring™ nCounter® transcriptomics and differential analysis of CDK9 inhibitors versus DMSO (0.1% (w/w)) on GCGR-E13 cells (n=3 per sample/condition). A. Network enrichment analysis (string-db.org) of NVP-2, filtered by minimum 3-fold change and FDR <0.05 (277 genes). B. Venn diagram comparison of differentially expressed genes across AZD4573, dinaciclib & NVP-2 with differentially expressed genes unique to NVP-2 and (NVP-2 + AZD4573) indicated. C. Summary of Biological processes (Gene Ontology) enriched in NVP-2 treated, GCGR-E13 cells (string-db.org). Coloured by FDR and sized by gene count.

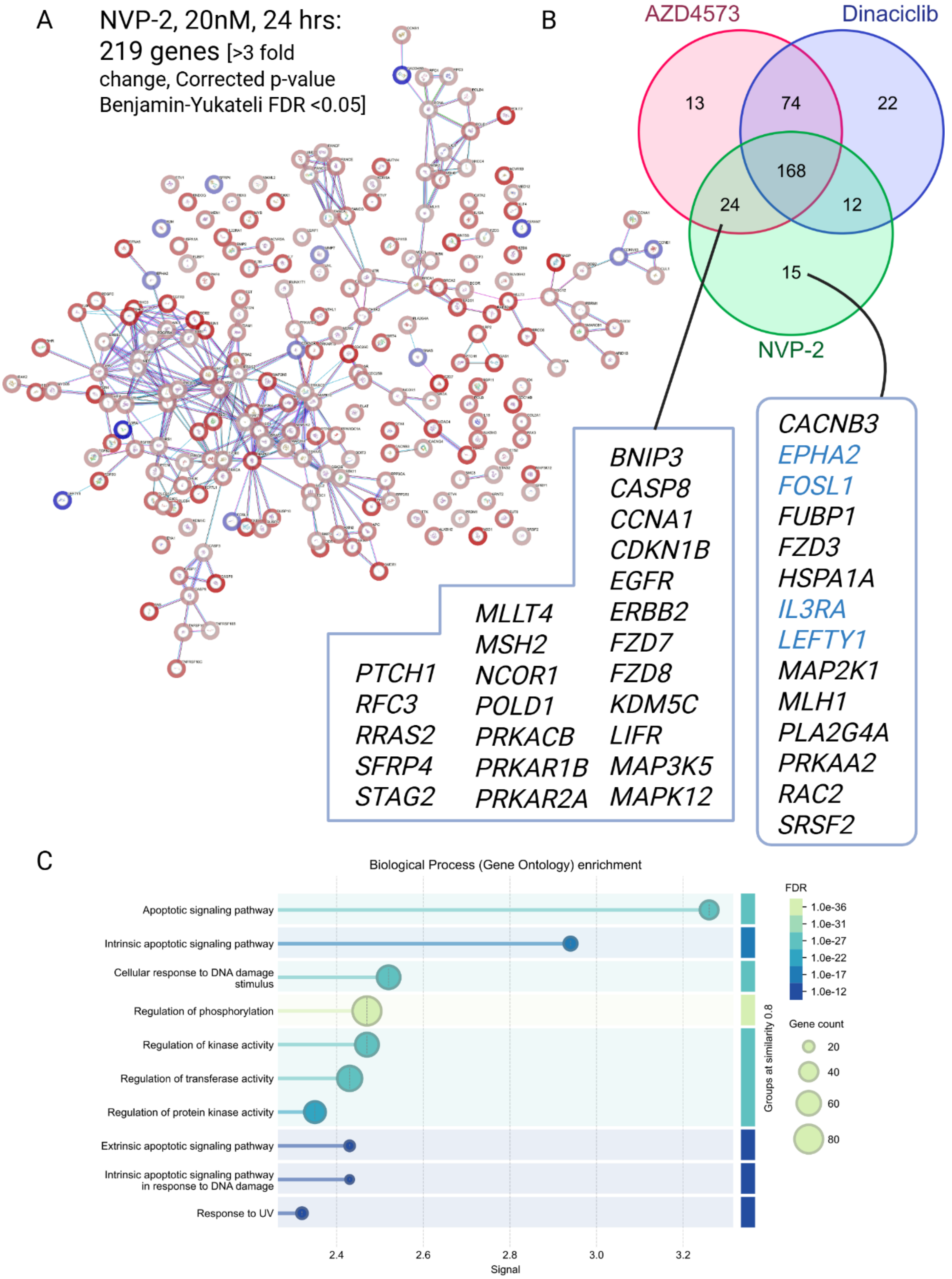

Supplementary Figure 12. Nanostring™ nCounter® transcriptomics and differential analysis of CDK9 inhibitors versus DMSO (0.1% (w/w)) on GCGR-E13 cells (n=3 per sample/condition). A. Network enrichment analysis (string-db.org) of dinaciclib, filtered by minimum 3-fold change and FDR <0.05 (277 genes). B. Venn diagram comparison of differentially expressed genes across AZD4573, dinaciclib & NVP-2 with differentially expressed genes unique to dinaciclib indicated. C. Summary of Biological processes (Gene Ontology) enriched in dinaciclib treated, GCGR-E13 cells (string-db.org). Coloured by FDR and sized by gene count.

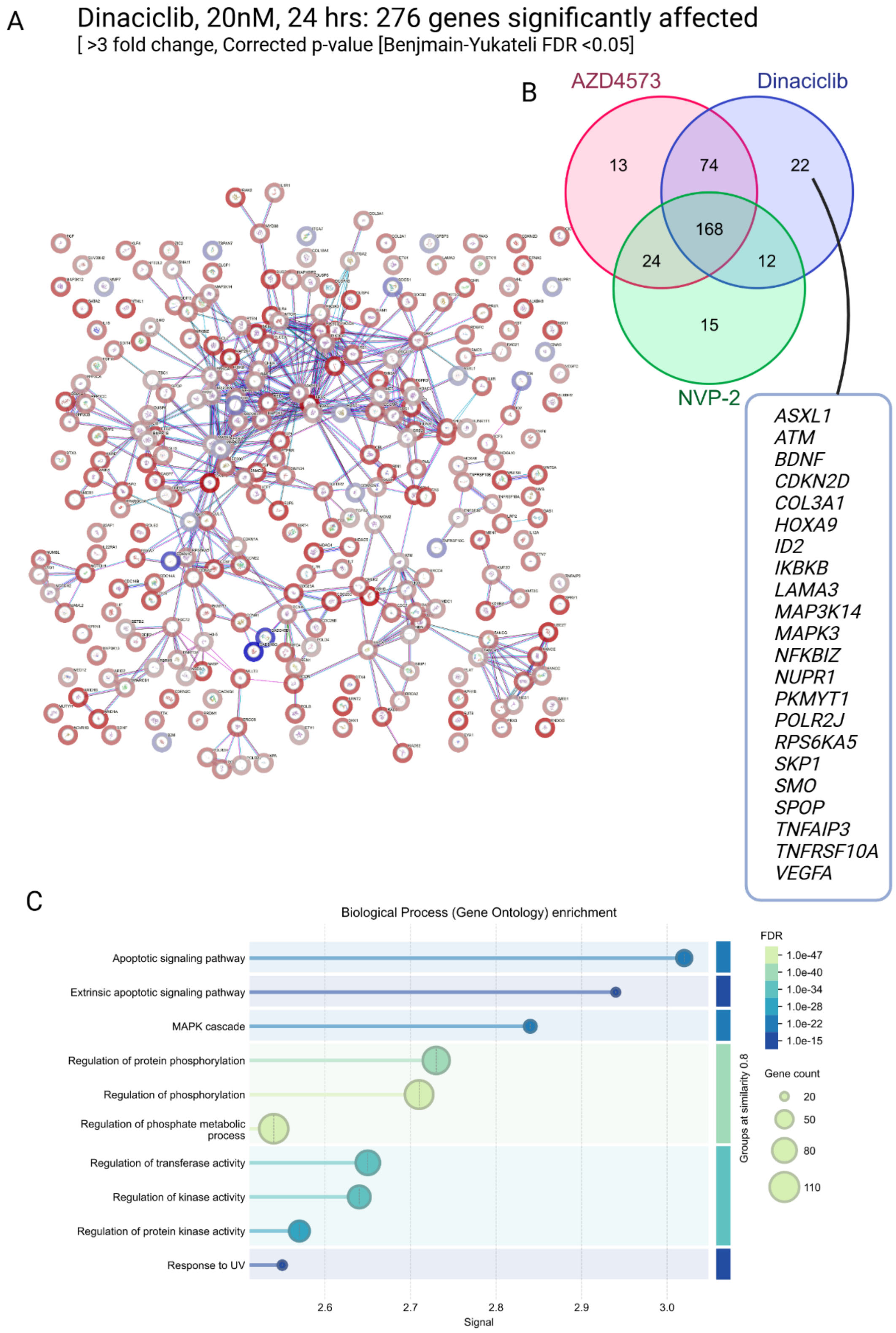

Supplementary Figure 13. Western blot analysis of dinaciclib, NVP-2 and AZD4573 (100nM) versus DMSO controls (n=4) across two GCGR cell lines, E13 and E57 using multiple probes. Cell lysates were prepared over two biological replicates at a 24 hour time point. A. Compound dependent loss of anti-apoptotic protein, MCL1, relative to DMSO controls (n=2). B&C. Modest compound effect on phospho-p38 but a reduction in total p38 MAPK was observed relative to DMSO controls. D&E. Compound dependent increase in ERK1/2 activity which inversely correlates with decrease in AKT activity (see panels K&L). *Note different exposures required between cell lines. F. Compound dependent loss of RAD51 expression in E13 cells (RHS), which is less pronounced in E57 cells (LHS). Arrows indicate RAD51 band for clarity. G. TSPAN7 levels were not affected by CDK9 inhibition at this 24 hour time point. (< non-specific band from M.W. ladder) H. Alpha-Tubulin loading control (''unequal loading on far right well is acknowledged but does not detract from the overall results/conclusions). [Panels I-M separate gel, same lysates used]. I. Compound dependent reduction of RNA polymerase II (arrows indicate RNAP2 band above non-specific bands in E57 cells). J. No change in BCL2 protein levels in E13 cells, which is very weakly expressed in E13 cells and was not detected in E57 cells. K. Compound dependent loss of AKT activity relative to DMSO wells and loading controls L & M (alpha-tubulin). *Note different exposures required between cell lines.

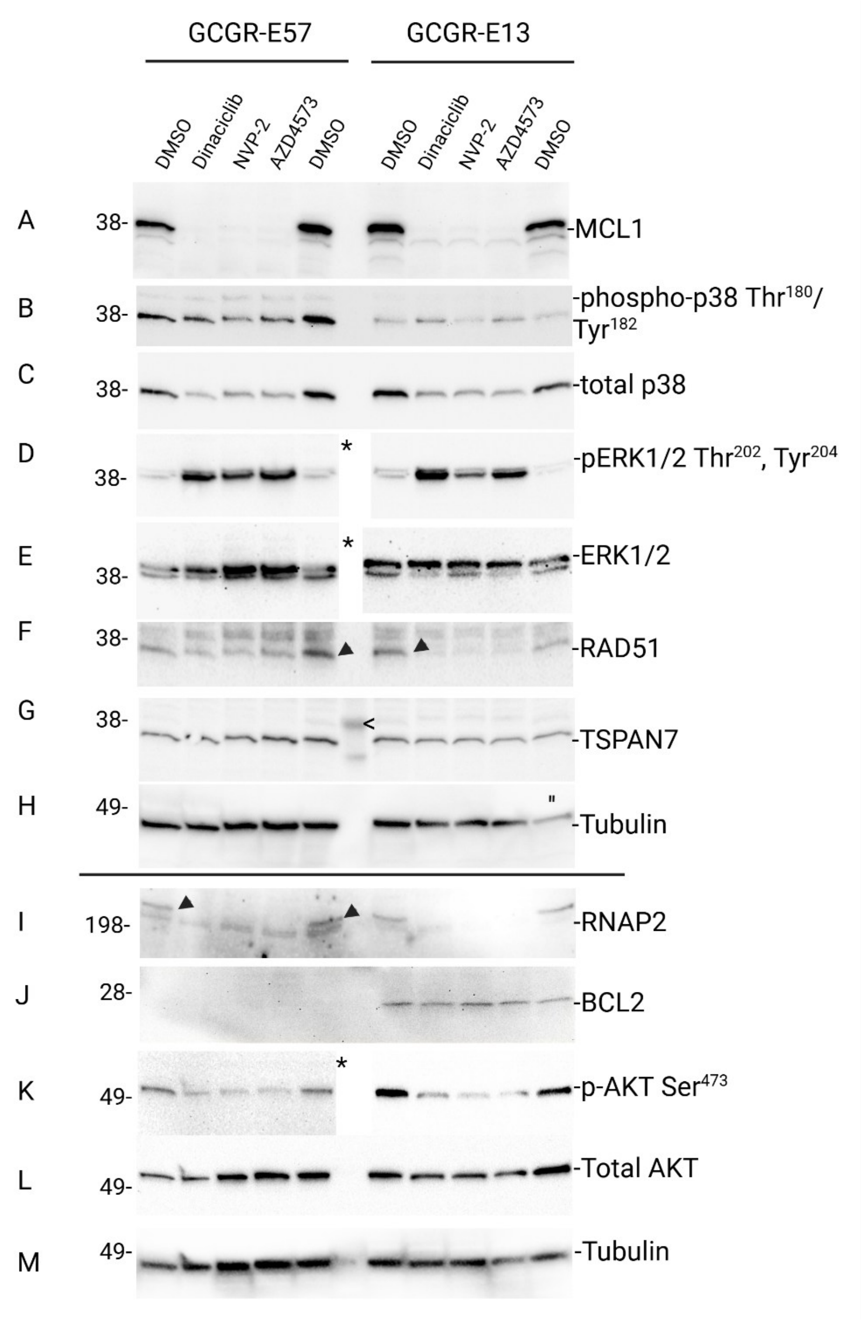

Supplementary Figure 14. Drug combination experiments between dinaciclib and romidepsin on GCGR-E13 cells. A. % cell survival data of romidepsin across GCGR cells by dose response (reproduced from figure 4, n=3, data points are represented as mean ± stdev).B. DepMap correlation predicts effective combination with dinaciclib and romidepsin (R^2 0.657) across multiple cancer lineages (PRISM Repurposing Public 24Q2 dataset, depmap.org). C. Representative images of dinaciclib x romidepsin combination matrix (Hoechst and phalloidin stain (green)). Scale bar 100um. D. Combination matrix (% Inhibition max = red) of dinaciclib (25 – 0.5nM) and romidepsin (10 – 0.1nM) E. 3D Synergy plot of dinaciclib x romidepsin matrix (synergy score 75% quantile <10: no synergy observed). 7x7 Synergy matrices were repeated twice comprising 36 dose-combination variations.

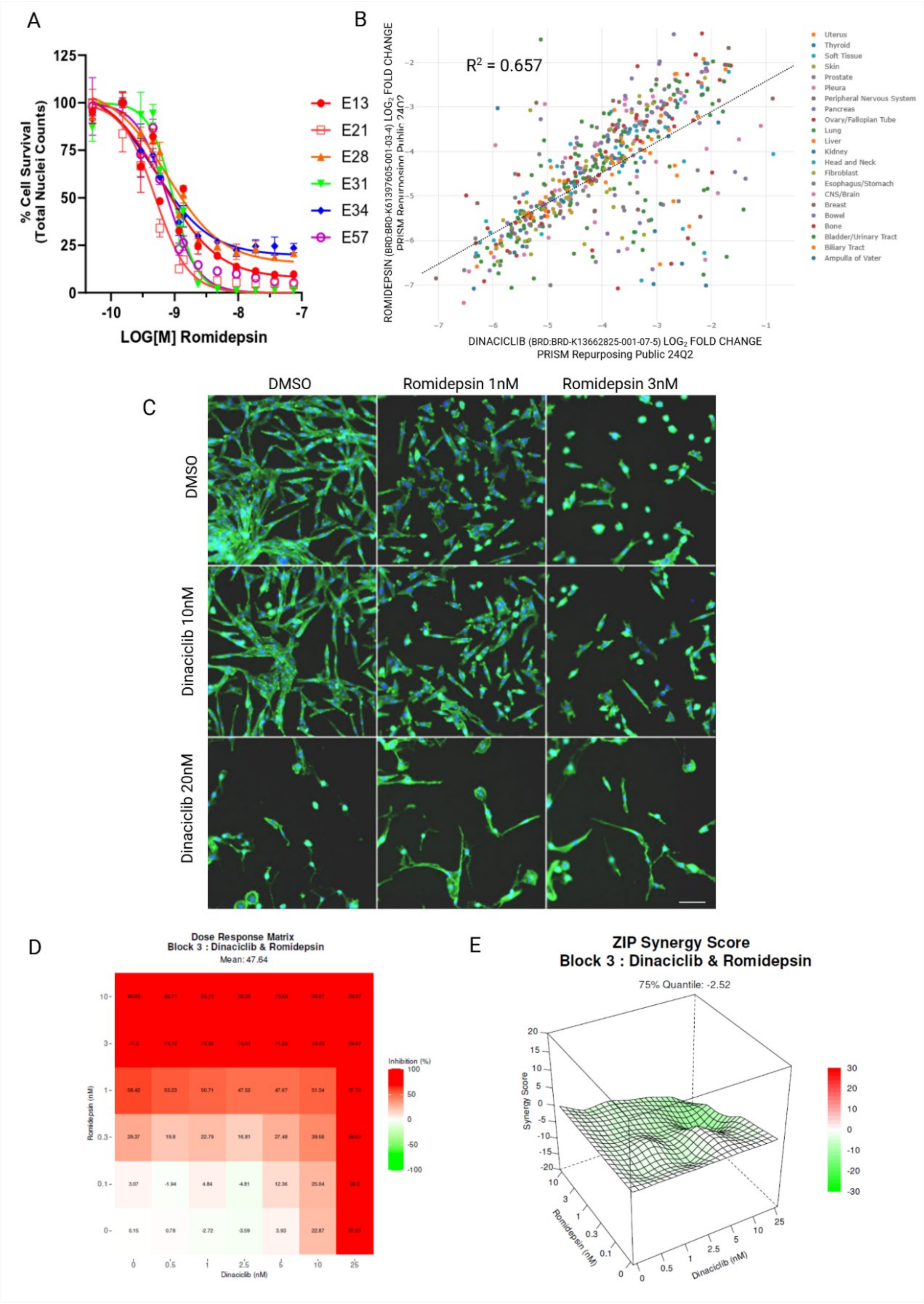

Supplementary Figure 15. Drug combination experiments between dinaciclib and idarubicin on GCGR-E13 cells. A. % cell survival data of idarubicin across GCGR cells by dose response (n=3, data points are represented as mean ± stdev). B. DepMap correlation predicts effective combination with dinaciclib and idarubicin (R^2 0.672) across multiple cancer lineages (PRISM Repurposing Public 24Q2 dataset, depmap.org). C. Representative images of dinaciclib x idarubicin combination matrix (Hoechst and phalloidin stain (green)). Scale bar 100um. D. Combination matrix (% Inhibition max = red) of dinaciclib (20 – 0.5nM) and idarubicin (80 – 1nM) E. 3D Synergy plot of dinaciclib x idarubicin matrix (synergy score 75% quantile <10: no synergy observed). 7x7 Synergy matrices were repeated twice comprising 36 dose-combination variations.

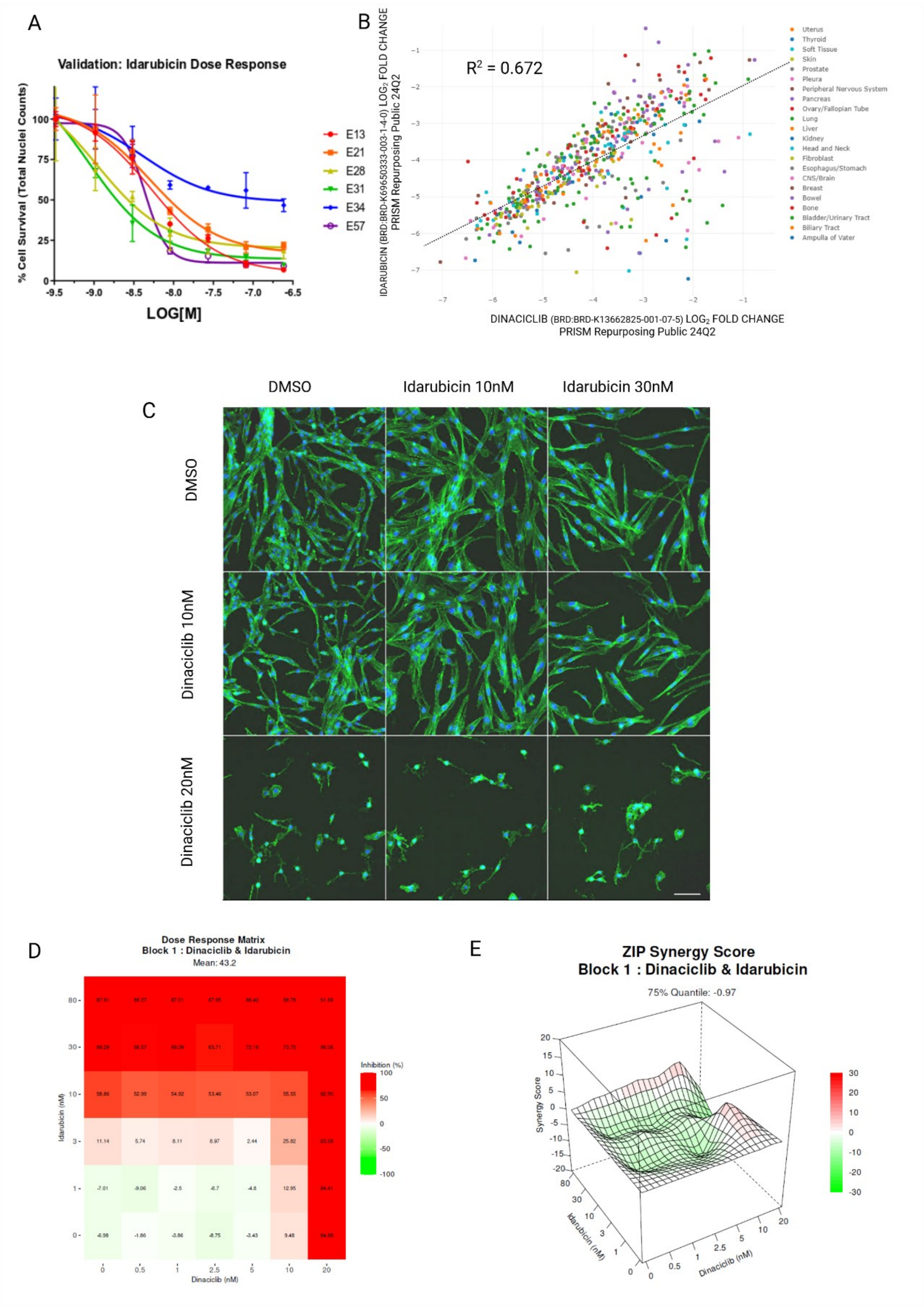

Supplementary Figure 16. Drug combination experiments between dinaciclib and ixazomib on GCGR-E13 cells. A. % cell survival data of ixazomib (citrate salt, MLN9708) across GCGR cells by dose response (n=3, data points are represented as mean ± stdev). B. DepMap correlation predicts effective combination with dinaciclib and ixazomib (citrate salt, MLN9708) (R^2 0.622) across multiple cancer lineages (PRISM Repurposing Public 24Q2 dataset, depmap.org). C. Representative images of dinaciclib x ixazomib (citrate salt, MLN9708) combination matrix (Hoechst and phalloidin stain (green)). Scale bar 100um. D. Combination matrix (% Inhibition max = red) of dinaciclib (25 – 0.5nM) and ixazomib (citrate salt, MLN9708) (80 – 1nM) E. 3D Synergy plot of dinaciclib x ixazomib matrix (synergy score 75% quantile <10: no synergy observed). 7x7 Synergy matrices were repeated twice comprising 36 dose-combination variations.

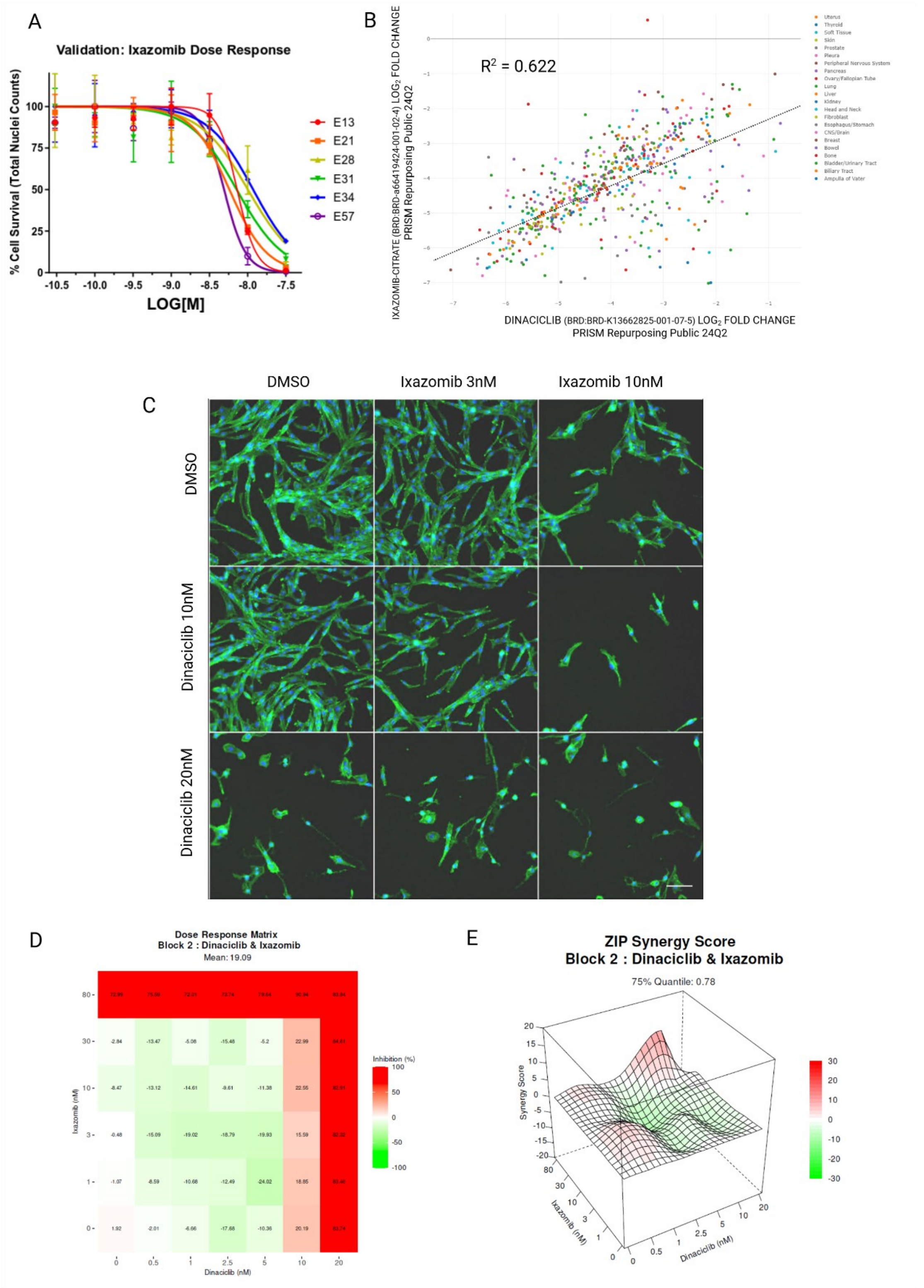

Supplementary Figure 17. Comparison of commercial, potent PI3K inhibitors on E13 cells. A. Live cell imaging of the effect of selective PI3K inhibitors versus dual PI3K/HDAC inhibitor, Fimepinostat (% confluence vs time (hrs) on GCGR-E13 cells (n=3, data points are represented as mean ± stdev). B. Summary table of published/quoted potencies (IC_{50} in nM) and selectivity across PI3K isoforms. Data reproduced over 3 biological replicates.

A

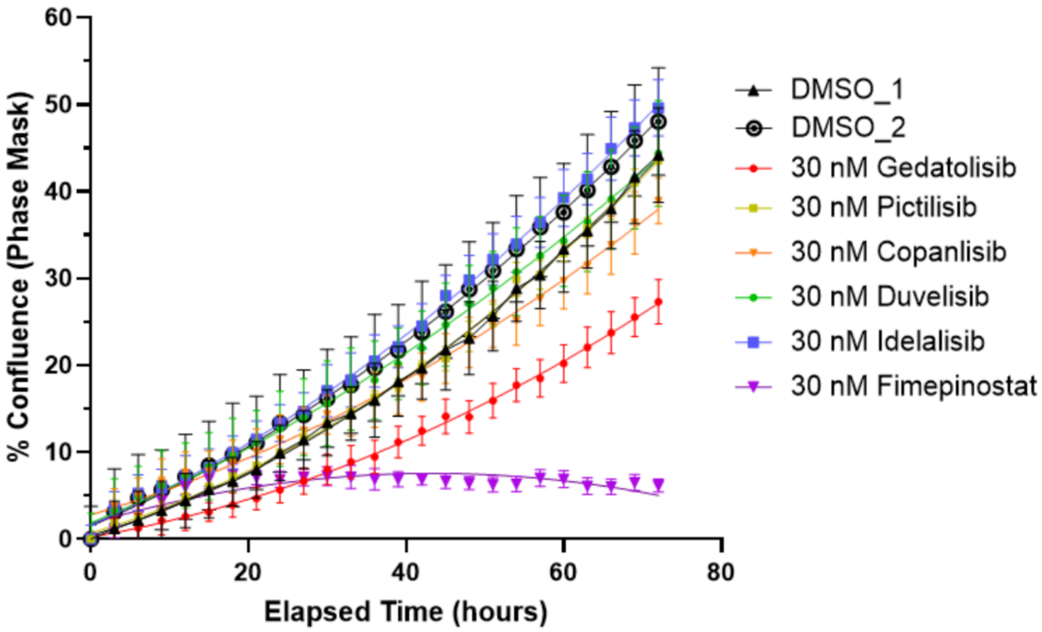

B

	In Vitro IC50 potency published 'cell free' data]									
Inhibitor/Target	PI3Kα	PI3Kβ	PI3Kγ	PI3Kδ	mTOR	HDAC1	HDAC2	HDAC3	HDAC10	HDAC11
Fimepinostat (CUDC-907)	19	54	311	39	-	1.7	5	1.8	2.8	5.4
Gedatolisib (PF-05212384)	0.4	-	5.4	-	1.6	-	-	-	-	-
Copanlisib (BAY 80-6946)	0.5	3.7	6.4	0.7	-	-	-	-	-	-
Pictilisib (GDC-0941)	3	33	75	3	-	-	-	-	-	-
Duvelisib (IPI-145)	1602	85	27	2.5	-	-	-	-	-	-
Idelalisib (CAL-101)	820	565	89	2.5	-	-	-	-	-	-

The IC_{50} selectivity data above is derived from the following literature:

DOIs:

Fimepinostat 10.1158/1078-0432.CCR-12-0055

Gedatolisib 10.1021/jm901830p

Copanlisib 10.1158/1535-7163.MCT-12-0993-T[1]

Pictillisib 10.1021/jm800295d

Duvelisib 10.1016/j.chembiol.2013.09.017

Idelalisib 10.1182/blood-2010-03-275305

Supplementary Table 1. List of validated compounds showing biological activity up to 1µM in two or more glioma stem cell lines. An electronic version of this table is also provided (see supplementary data). ** Indicates compounds which are predicted (or otherwise known) to be CNS/Brain penetrant (74 of 164 compounds = 45% BBB prediction).Shaded GREEN: priority group 1 compounds, Shaded ORANGE (priority group 2) and shaded RED: deprioritised compounds.

ACTIVE [validated] COMPOUNDS (2+ CELL LINES)

Prioritised_group1	#	*Compound ID*	*Target(s)*	#	*Compound ID*	*Target(s)*	#	*Compound ID*	*Target(s)*
Prioritised_group2 (follow up)	1	Carmofur**	5-FU, antimetabolite	56	Belinostat**	HDAC (pan)	111	Nitidine**	ND
De-prirotised	2	Dasatinib	ABL, SRC, c-KIT	57	Dacinostat**	HDAC (pan)	112	OTS514	NEK1, TOPK
74/164 (45%) BBB prediction	3	Bosutinib**	ABL, SRC, STAT3, ERK	58	Oxamflatin**	HDAC (pan)	113	Auranofin**	NFkB/IL6-STAT3
** BBB permeable (predicted)	4	TIC10 angular**	AKT/ERK	59	Panobinostat**	HDAC (pan)	114	Antimycin A**	OX PHOS
or known BBB pentrant	5	Lorlatinib	ALK, ROS1	60	Scriptaid**	HDAC (pan)	115	PF-03758309	PAK4/6 (pan-PAK)
	6	Crizotinib	ALK/ROS1	61	M-344	HDAC (pan)	116	AZD5305/Saruparib	PARP1
	7	Silodosin	alphaA1-AR	62	Trichostatin A	HDAC (pan)	117	olaparib**	PARP1/2
	8	Tosedostat	aminopeptidase	63	CUDC-101**	HDAC (pan), EGFR, HER2	118	Rucaparib**	PARP1/2
	9	Tosufloxacin hydrochlori	antibiotic	64	Quisinostat**	HDAC1	119	Talazoparib**	PARP1/2
	10	Calcimycin	antibiotic, Ca ionophore	65	Romidepsin	HDAC1,2	120	Niraparib**	PARP1/2
	11	Miconazole**	antifungal	66	Tacedinaline**	HDAC1,2,3,8	121	Phorbol 12-myristate 13-acetate	PKC agonist
	12	Sulconazole nitrate**	antifungal	67	CUDC-907 (fimepinostat)	HDAC1/2/3/6/10/11 & PI3K	122	staurosporine	PKC, pan-kinase
	13	Fenbendazole**	antihelminth	68	Mocetinostat	HDAC2,3,11	123	U-73343**	PLC, 5-LO
	14	Flubendazole**	antihelminth	69	LMK-235**	HDAC4,5	124	Volasertib	PLK1
	15	Ciclesonide**	anti-inflammatory	70	Emetine dihydrochloride	HIF, PDK1, BRD4, ROCK	125	GSK269962	PRKCI /ROCK
	16	Azaguanine-8**	antimetabolite	71	NXP-800**	HSF-1	126	Ixazomib**	Proteasome
	17	Ancitabine**	anti-metabolite	72	CCT251236	HSF1, PIR	127	Bortezomib	Proteasome
	18	Cyclocytidine**	anti-metabolite	73	Ganetespib	HSP90	128	Carfilzomib	Proteasome
	19	Cytarabine**	anti-metabolite	74	NMS-E973**	HSP90AA1	129	Cycloheximide**	Protein Synthesis
	20	Gemcitabine HCl	anti-metabolite	75	GSK1838705A	IGF-1R	130	Homoharringtonine**	Protein Translation
	21	Methyl benzethonium c	anti-microbial	76	Bay 11-7082**	IKK	131	LY-411575	PSEN
	22	Thioridazine**	anti-psychotic	77	Momelotinib	JAK1/2	132	Alexidine**	PTPMT1
	23	Alisertib	AURK	78	Gandotinib	JAK2	133	Retinoic acid p-hydroxyanilide**	RAR
	24	Aurora-A Inhibitor I	AURK	79	Pacritinib	JAK2/1, FLT3	134	Clofarabine**	RNR, DNA Pol
	25	CCT137690	AURK	80	Hexachlorophene**	KCNQ1/KCNE1 K channel activa	135	GSK650394	SGK
	26	Tozasertib	AURK	81	SB 743921	KIF11/KSP	136	AZD3965	SLC16A1 (MCT1)
	27	GSK1070916	AURK/SIK	82	GDC-0623	MEK1	137	Ponatinib**	SRC, ABL, PDGFR, VEGFR, F
	28	AZD5582	BIRC3	83	Trametinib	MEK1/2	138	AC-93253 iodide**	SRC, EGFR, FAK
	29	GSK-1324726A (I-BET72	BRD2/3/4	84	OTSSP167	MELK	139	eCF506/NXP-900**	SRC/FYN/YES
	30	GSK1210151A	BRD4	85	Cabazitaxel	Micotubule	140	Saracatinib**	SRC/FYN/YES
	31	Flavopiridol	CDK1/2/4/6/9	86	Albendazole**	Microtubule	141	PF-429242**	SREBP S1 protease
	32	Dinaciclib	CDK1/2/5/9	87	Cantharidin**	Microtubule	142	Stattic	STAT3
	33	AZD5438	CDK1/2/9	88	Mebendazole**	Microtubule	143	Niclosamide**	STAT3
	34	Abemaciclib**	CDK4/6	89	Nocodazole**	Microtubule	144	Pyrvinium pamoate	STAT3, Wnt
	35	Palbociclib	CDK4/6	90	Podophyllotoxin**	Microtubule	145	Thonzonium bromide	surfactant
	36	AZD4573	CDK9	91	Suprafenacine**	Microtubule	146	Chaetocin*8	SUV39H1 (KMT1A)
	37	NVP-2	CDK9/CyclinT1	92	Thiocolchicine**	Microtubule	147	Irinotecan**	TOPOI
	38	Foretinib**	c-MET, KDR, FLT4	93	2-methoxyestradiol	Microtubule	148	Hydroxy Camptothecine	TOPOI
	39	Brequinar sodium salt hy	DHODH	94	Cephalomannine	Microtubule	149	Ellipticine**	TOPOII
	40	Pipobroman**	DNA alkylation	95	Colchicine	Microtubule	150	Amsacrine	TOPOII
	41	5-azacytidine**	DNA methylation	96	Docetaxel	Microtubule	151	Daunorubicin hydrochloride	TOPOII
	42	PD153035**	EGFR	97	Fosbretabulin Disodium	Microtubule	152	Doxorubicin hydrochloride	TOPOII
	43	Olmutinib	EGFR	98	Ixabepilone	Microtubule	153	Etoposide**	TOPOII
	44	Osimertinib mesylate	EGFR T790M	99	paclitaxel	microtubule	154	Idarubicin	TOPOII
	45	CR-1-31B**	EIF4A (protein Translatio	100	Vinblastine sulfate	Microtubule	155	Mitoxantrone	TOPOII
	46	Rotenone**	Electron Transport	101	Vinorelbine Tartrate	Microtubule	156	Pirarubicin	TOPOII
	47	POM-HEX**	ENOL2/1	102	Torin2**	mTOR	157	Pixantrone dimaleate	TOPOII
	48	Selinexor**	exportin-1 (XPO1)	103	Sapanisertib	mTOR (kinase)	158	Teniposide	TOPOII
	49	VS-4718	FAK (PTK2), PYK2	104	Gedatolisib	mTOR/PI3K	159	Topotecan	TOPOII
	50	GSK2194069	FASN	105	Digoxigenin	Na/K ATPase	160	Piperlongumine**	TrxR1, PI3K/mTOR
	51	Simvastatin**	Ferroptosis agonist	106	Digoxin	Na/K-ATPase	161	ML240	VCP/p97
	52	BAY-876	GLUT1	107	Lanatoside C	Na/K-ATPase	162	Anlotinib**	VEGFR2/3, c-KIT
	53	BIO [6-BROMOINDIRUB	GSK-3	108	MG 624	nAChR	163	CID 11210285**	WNT agonist
	54	Givinostat	HD1A	109	Diphenyleneiodonium chl	NADPH oxidsae	164	YAP-TAZ-INH1	YAP/TAZ

Supplementary Table 2. [*HTS validation and QC: see separate excel file*]
Supplementary Table 3. [*Validation – enrichment table from network analysis: see separate excel file*]
Supplementary Table 4. [Similarity matrix – RPPA/Cytokine/IC50 data: *see separate excel file*]
Supplementary Table 5. [CDK9 inhibitors Nanostring data and STRING/enrichment data*: see separate excel file*]
Supplementary Table 6. [Compounds purchased for validation*: see separate excel file*]
Supplementary Table 7. [PCA factor loadings from dimensionality reduction/reduction/principal component analysis *: see separate excel file*]

REFERENCES [Supplementary Information]

[1] Wang Q, Hu B, Hu X, Kim H, Squatrito M, Scarpace L, deCarvalho AC, Lyu S, Li P, Li Y, Barthel F, Cho HJ, Lin YH, Satani N, Martinez-Ledesma E, Zheng S, Chang E, Sauvé CG, Olar A, Lan ZD, Finocchiaro G, Phillips JJ, Berger MS, Gabrusiewicz KR, Wang G, Eskilsson E, Hu J, Mikkelsen T, DePinho RA, Muller F, Heimberger AB, Sulman EP, Nam DH, Verhaak RGW. Tumor Evolution of Glioma-Intrinsic Gene Expression Subtypes Associates with Immunological Changes in the Microenvironment. Cancer Cell. 2017 Jul 10;32(1):42-56.e6. doi: 10.1016/j.ccell.2017.06.003. Erratum in: Cancer Cell. 2018 Jan 8;33(1):152. doi: 10.1016/j.ccell.2017.12.012. PMID: 28697342; PMCID: PMC5599156.

[2] Richards LM, Whitley OKN, MacLeod G, Cavalli FMG, Coutinho FJ, Jaramillo JE, Svergun N, Riverin M, Croucher DC, Kushida M, Yu K, Guilhamon P, Rastegar N, Ahmadi M, Bhatti JK, Bozek DA, Li N, Lee L, Che C, Luis E, Park NI, Xu Z, Ketela T, Moore RA, Marra MA, Spears J, Cusimano MD, Das S, Bernstein M, Haibe-Kains B, Lupien M, Luchman HA, Weiss S, Angers S, Dirks PB, Bader GD, Pugh TJ. Gradient of Developmental and Injury Response transcriptional states defines functional vulnerabilities underpinning glioblastoma heterogeneity. Nat Cancer. 2021 Feb;2(2):157-173. doi: 10.1038/s43018-020-00154-9. Epub 2021 Jan 4. PMID: 35122077.
